# The RSF1 Histone-Remodelling Factor Facilitates DNA Double-Strand Break Repair by Recruiting Centromeric and Fanconi Anaemia Proteins

**DOI:** 10.1371/journal.pbio.1001856

**Published:** 2014-05-06

**Authors:** Fabio Pessina, Noel F. Lowndes

**Affiliations:** Genome Stability Laboratory, Centre for Chromosome Biology, School of Natural Science, National University of Ireland Galway, Ireland; Dana-Farber Cancer Institute, United States of America

## Abstract

ATM is a central regulator of the cellular responses to DNA double-strand breaks (DSBs). Here we identify a biochemical interaction between ATM and RSF1 and we characterise the role of RSF1 in this response. The ATM–RSF1 interaction is dependent upon both DSBs and ATM kinase activity. Together with SNF2H/SMARCA5, RSF1 forms the RSF chromatin-remodelling complex. Although RSF1 is specific to the RSF complex, SNF2H/SMARCA5 is a catalytic subunit of several other chromatin-remodelling complexes. Although not required for checkpoint signalling, RSF1 is required for efficient repair of DSBs via both end-joining and homology-directed repair. Specifically, the ATM-dependent recruitment to sites of DSBs of the histone fold proteins CENPS/MHF1 and CENPX/MHF2, previously identified at centromeres, is RSF1-dependent. In turn these proteins recruit and regulate the mono-ubiquitination of the Fanconi Anaemia proteins FANCD2 and FANCI. We propose that by depositing CENPS/MHF1 and CENPX/MHF2, the RSF complex either directly or indirectly contributes to the reorganisation of chromatin around DSBs that is required for efficient DNA repair.

## Abstract 2

RSF1, a new player in the cellular responses to DNA double-strand breaks, sequentially recruits centromeric histone-like proteins and DNA repair proteins from the Fanconi anaemia pathway.

## Author Summary

DNA carries all the information necessary for life; thus damage or loss of genetic material can result in cell death or cancer. The worst-case insult to genetic information is a DNA double-strand break, caused by agents either within the cell (e.g., by-products of respiration, errors of DNA replication) or from outside (e.g., ionizing radiation). Ataxia telangiectasia kinase (ATM) and the Fanconi anaemia proteins perform housekeeping roles in the cell, recognising aberrant DNA structures and promoting their repair. Mutations that affect these proteins are responsible for the eponymous genetic syndromes that are characterised by elevated mutation rate, increased cancer risk, developmental defects, and shortened life span. In this work we identify and characterise a novel link between these two central players in the DNA damage response. We show that the Remodelling and Spacing Factor 1 (RSF1) protein, which can reorganise the compaction of DNA to allow access for other proteins, requires ATM for its function after DNA damage. Specifically, RSF1 recruits two centromeric histone-like proteins that in turn promote mono-ubiquitination and recruitment to sites of damage of FANCD2 and FANCI—two proteins that belong to the Fanconi anaemia pathway. Absence of RSF1 results in defective repair of double-strand DNA breaks, prolonged arrest of the cell cycle, and cell death. Our study suggests that ATM-dependent regulation of the RSF chromatin-remodelling complex is necessary during double-strand break repair to recruit centromeric histones and then Fanconi anaemia proteins.

## Introduction

DNA damage can result in mutations leading to either cell death or cancer, and multiple repair pathways exist that are specific to distinct DNA lesions [1],[2]. DNA double-strand breaks (DSBs) are particularly toxic lesions repaired by two major pathways, termed homologous recombination (HR) or nonhomologous end joining (NHEJ), that utilise either homology-dependent or -independent mechanisms. Additional biological responses to DNA damage include altered transcriptional programmes, transient cell cycle delays termed checkpoints, apoptosis, and senescence. Collectively these responses are termed the DNA damage response (DDR).

Ataxia telangiectasia, mutated (ATM) and ATM and Rad3-related (ATR), a pair of related protein kinases, are central to the DDR [3]. ATM is directly recruited to DSBs via the Mre11–Rad50–NBS1 (MRN) complex, whereas ATR, with its partner ATR-interacting protein (ATRIP), is indirectly recruited via the single-stranded DNA (ssDNA) generated during DSB processing. ATM and ATR initiate signalling cascades by phosphorylating many target proteins, including checkpoint kinase 1 and 2 (Chk1 and Chk2), which initiate a secondary wave of phosphorylation events. Additional posttranslation modifications, including ubiquitinylation, SUMOylation, poly(ADP-ribosylation), acetylation, and methylation, are also required for a successful DDR.

DNA is packaged around the core histone proteins H2A, H2B, H3, and H4 to form nucleosomes and nucleosomes in turn interact with many other nonhistone proteins to form chromatin, which must be dynamically remodelled for a successful DDR [4]. Remodelling of chromatin requires a multitude of chromatin remodelling enzymes and encompasses not only nucleosome removal or sliding but also modification of core histones or their replacement by histone variants. For example, SNF2H (also termed SMARCA5) is an ATP-dependent translocase that is the catalytic component of at least four chromatin-remodelling complexes. These include (1) the ACF/WCRF complex composed of SNF2H and the ACF/WCRF protein, also known as BAZ1A [5]; (2) the CHRAC complex composed of SNF2H and the CHRAC1, POLE3, and ACF1 proteins [6]; (3) the RSF complex composed of SNF2H and RSF1 [7],[8]; and (4) the WICH complex composed of SNF2H and the BAZ1B, DEK, DDX21, ERCC6, MYBBP1A, and SF3B1 proteins [9].

An important connection between the ATM and ATR kinases and the ACF/WCRF, CHRAC, RSF, and WICH complexes has been established in a screen for substrates of these kinases [10]. Additionally, the ACF/WCRF complex is required for DSB repair [5] and the WICH complex has been implicated in the DDR [11]. More recently, the BAZ1B component of the WICH complex was shown to be required for the activation of ATM and phosphorylation of H2AX at tyrosine residue 142 [12].

Although a direct connection to DNA repair has yet to be made for the RSF1 protein, it is required for the stabilisation of the CENPA complex and correct assembly of centromeric chromatin [7],[8]. Interestingly, the CENPS–CENPX centromeric chromatin complex, composed of histone fold proteins that most resemble histones H3 and H4, has recently been shown to participate in the repair of DNA interstrand cross-links (ICLs) via the Fanconi Anaemia (FA) pathway [13],[14]. CENPS and CENPX have also been termed MHF1 and MHF2 for FANCM-interacting Histone Fold protein 1 and 2. Additionally, overexpression of RSF1 in an ovarian cancer cell line has also been reported to induce DNA damage and genomic instability [15].

Patients with FA are defective in a pathway required for the repair of DNA ICLs, causing elevated genome instability and cancer risk [16]–[18]. The FA pathway regulates the mono-ubiquitination of two related proteins, FANCD2 and FANCI, that in turn coordinate the recruitment of several nucleases required to unhook the ICL, resulting in an unhooked cross-linked nucleotide on one DNA molecule and a DSB on the other. Translesion synthesis (TLS) allows bypass of the unhooked cross-linked nucleotide, necessary for generating a template suitable for HR-dependent DSB repair. Nucleotide excision repair (NER) then removes the nucleotide with the cross-linked adduct. Initiation of the FA pathway requires the multifunctional FANCM–FAAP24 heterodimer, which recognises ICLs, recruits the FA core complex, and contributes to both stabilisation of stalled replication and ATR-dependent signalling.

We used a proteomic screen to identify proteins that interact with chicken Atm and focussed on Remodelling and Spacing Factor 1 (Rsf1), a subunit of a chromatin-remodelling factor. We show that human RSF1 and ATM directly interact and map the regions of interaction between these proteins. RSF1 has been identified as a likely ATM substrate after ionising radiation (IR) [10], although its role in the DDR had not been characterised. We have defined a role for human RSF1 in the repair of DNA DSBs by both NHEJ and HR. Interestingly, the RSF1 protein directly interacts with the histone-related CENPS/MHF1 and CENPX/MHF2 proteins, previously implicated in centromere function and the FA pathway. We find that RSF1 is required for localization of the CENPS/MHF1–CENPX/MHF2 complex, followed by FANCD2 and FANCI to sites of DNA damage. Similarly, RSF1 is required for the efficient mono-ubiquitination and chromatin retention of FANCD2 and FANCI after IR. Our data suggest that ATM-dependent regulation of the RSF chromatin-remodelling complex is required during DSB repair for the sequential recruitment of centromeric and FA proteins to facilitate efficient DSB repair.

## Results

### Generation of the HFSC-*Atm* Cell Line

**Figure 1:**
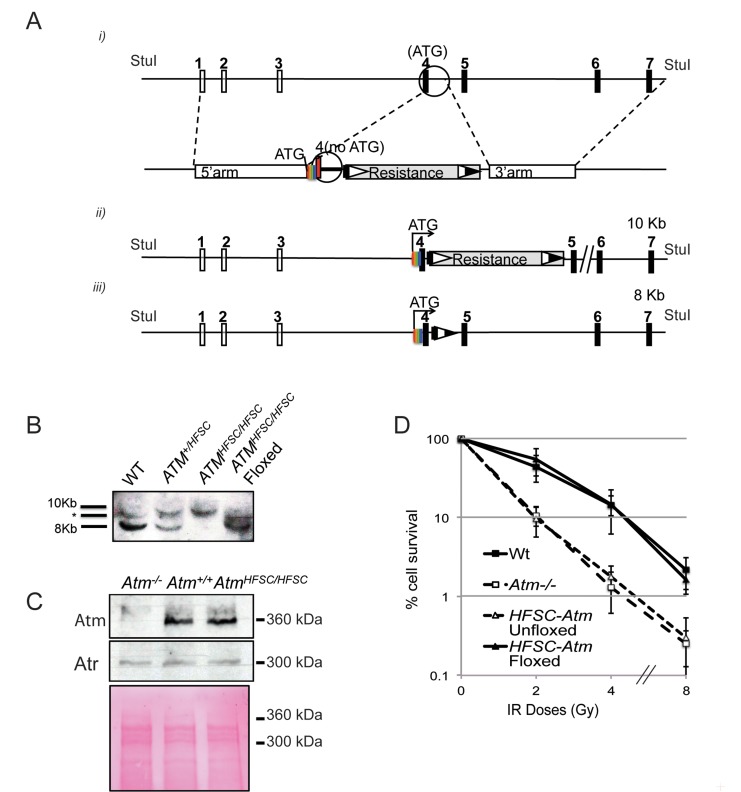
Targeting the *Atm* locus to generate the HFSC–Atm cell line. (A) Schematic of the chicken *Atm* locus with noncoding and coding exons indicated by open and filled rectangles, respectively. (*i*) Knock-in strategy, the 5′ targeting arm spans from the first noncoding exon to the first coding exon. The HFSC tag was synthesized in frame with the *Atm* genomic sequence, excluding the endogenous ATG, and extending into the intron between the first two coding exons. It was fused to the 5′ targeting arm by PCR and cloned into the targeting vector. The 3′ targeting arm commences precisely where the synthesized DNA ended and extends to beyond the fourth coding exon. (*ii*) Structure of the *Atm* locus after targeting and before floxing. (*iii*) Structure of the *Atm* locus after floxing. The StuI sites indicate the DNA fragments detected by Southern blot analysis, corresponding to ∼8 kb for both the WT locus and the floxed locus and to ∼10 kb for the locus after gene targeting by prior to floxing. (B) Southern blot demonstrating successful gene targeting (the asterisk indicates an unspecific band). (C) Western blot demonstrating normal expression of HFSC–Atm in the indicated cell lines. (D) Clonogenic survival analyses of the indicated cell lines at the indicated IR doses.

We used a novel HFSC-affinity tag (Figure S1A) together with gene targeting in chicken DT40 cells (Figure 1A) to generate a cell line expressing HFSC-Atm as the sole source of the chicken Atm protein. Southern blotting confirms that both *Atm* alleles were successfully targeted using a strategy that results in minimal genetic alteration to the *Atm* locus (Figure 1A and 1B). Expression from the WT *Atm* promoter produces HFSC-Atm at WT levels (Figure 1C). Clonogenic survival assays confirmed that HFSC-*Atm* cells survived IR as well as WT cells (Figure 1D).

SILAC quantitative proteomic analyses both before and after IR were performed to identify Atm-interacting proteins. Known ATM-interacting proteins were identified (Table 1 and see Table S1 for a full list of the proteins identified). We noticed that several chicken chromatin-remodelling factors were enriched in our screen, including Rsf1, a protein without a characterized role in the DDR.

**Table 1:** Previously identified ATM-interacting proteins and SNF2H-related chromatin remodelling factors identified in the screen.

Gene Name	Protein ID	− IR	+ IR	Uniprot	Peptide	Mol Weight (kDa)	Sequence Length	PEP	Reference
ATM	IPI00601074	18.0345	17.871		130	349.74	3,069	0	[44]
USP10	IPI00819631	1.327425	1.281195	Q5ZJN4	25	87.558	795	1.48E-127	[45]
FANCI	IPI00586876			B0I564	1	149.29	1,338	0.018182	[46]
MCM3	IPI00604115	1.146465	1.471155	Q5ZMN2	27	91.25	812	7.62E-123	[47]
MCM2	IPI00577675	1.12		Q5ZLZ1	22	100.35	888	1.11E-130	[48]
PPP2A	IPI00589014	1.01		P48463	3	35.563	309	8.39E-16	[49]
CHD4	IPI00598956	1.2	1.65		11	215.81	1,895	7.86E-38	[50]
RFC1	IPI00683437			Q5ZKU2	1	129.19	1,153	0.001435	[20]
PCNA	IPI00821596	1.130685		Q9DEA3	14	29.016	263	2.69E-109	[51]
RSF1	IPI00572615		1.44654		4	163.86	1,441	6.55E-11	N/D
SNF2H	IPI00577188		1.39305		21	136.85	1,198	2.36E-134	N/D
BAZ1A	IPI00585200		1.35342		2	180.23	1,571	1.85E-05	N/D
BAZ1B	IPI00820657				1	164.02	1,483	5.55E-22	N/D

Gene name, protein ID, the relative enrichment ratios compared to an untagged cell line of the un-irradiated and irradiated samples, the Uniprot identifier (if known), the number of peptides identified, the molecular weight of the identified protein, the number of its amino acids, and PEP score are indicated. Proteins previously identified as ATM-interacting proteins are indicated by the relevant reference.

### RSF1 Interaction with ATM Is Dependent upon IR and ATM Kinase Activity

**Figure 2:**
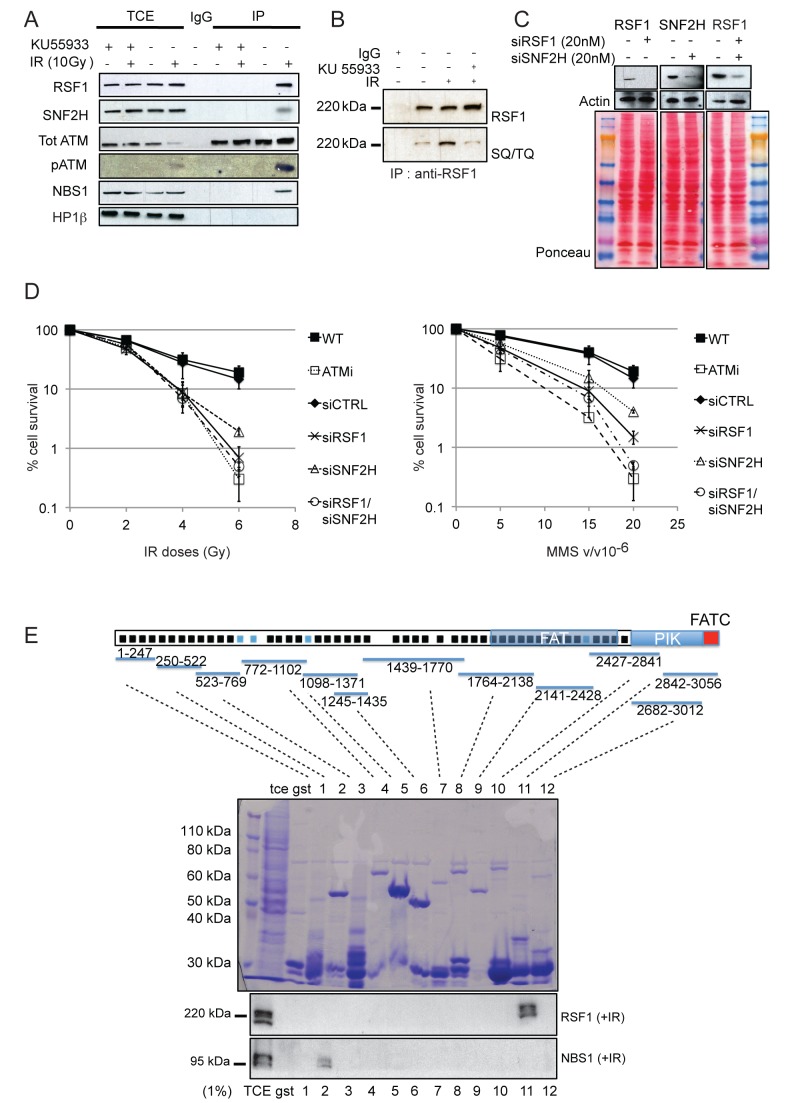
RSF1 is a novel ATM-interacting protein required for cell survival after DNA damage. (A) Co-immunoprecipitation (note that benzonase was included during cell lysis to solubilise chromatin-bound proteins; see Materials and Methods) of the indicated proteins with ATM. U2OS cells were either mock treated or treated with 10 Gy IR and harvested 1 h after irradiation. Where indicated the ATM inhibitor KU55933 was added directly to the media an hour before irradiation. (B) RSF1 immunoprecipitation. Cells were mock treated or treated with 5 Gy of IR and 5 Gy of IR plus ATM inhibitor. Elutions were blotted with RSF1 monoclonal antibody (Millipore) and general SQ/TQ antibody (Cell Signalling). (C) Typical efficiency of RSF1 and SNF2H depletion (siRNA) in U2OS cells. (D) Clonogenic survival after the indicated treatments of U2OS cells with IR or MMS. (E) Recombinant GST fusion proteins expressing fragments of ATM proteins were purified by *E. coli*, and approximately 5 mg of each GST fusion protein was used to incubate with cell lysates from HEK293 cells that had been previously irradiated with 5 Gy of IR and harvested 1 h post-IR. Elutions were blotted for RSF1 together with NBS1 as a control [37]. Schematic adapted from [43] shows heat repeats (black squares) conserved in the PIK family and heat repeats specific for ATM protein (blue squares); the domains are indicated, as are the regions where the GST fragments are mapping.

We validated the chicken Atm–Rsf1 complex by co-immunoprecipitation of human RSF1 with human ATM using U2OS osteosarcoma cells (Figure 2A). Although a well-characterised DNA-damage–dependent ATM interactor, NBS1 [19],[20], co-immunoprecipitated with ATM after IR, HP1β, an abundant chromatin protein, also implicated in the DDR [21], did not. RSF1, although chromatin-bound both before and after IR (Figure S2F), only co-immunoprecipitated with ATM after IR. Similarly, the SNF2H (or SMARCA5) catalytic subunit of the RSF complex also co-immunoprecipited with ATM after IR. The interaction between ATM and the RSF complex is not only dependent upon IR but also upon ATM kinase activity (Figure 2A) and could also be detected in extracts from HEK293 human embryonic kidney cells (Figure S2A and S2B). The equivalent interaction detected between chicken Atm and both Rsf1 and Snf2H/Smarc5 was also IR-dependent (Table 1).

RSF1 has been reported to be a substrate of ATM *in vitro* [10], and here we show that RSF1 phosphorylation *in vivo* is also ATM-dependent (Figure 2B). Additionally, using the anti-pS1423BRCA1 antibody used by Matsuoka and colleagues [10] to detect phosphorylation of additional ATM/ATR substrates, we show that phosphorylation of RSF1 is largely ATM-dependent (Figure S2G). Moreover, as the C-terminal consensus PIK kinase sites more closely match the BRCA1-S1423 epitope, our data suggest that it is these two C-terminal sites, rather than the N-terminal consensus PIK kinase site, that is phosphorylated *in vivo*.

Human SNF2H is also a component of the ACF1/WRCF, CHRAC, and WICH chromatin remodelling complexes, whereas RSF1 is specific to the RSF complex alone [7],[8]. Of note, the BAZ1A (also termed ACF1) and BAZ1B components of the ACF/WCRF and WICH complexes, respectively, were also identified in our human ATM immunoprecipitates (Figure S2A, S2B, and S2C) and their chicken homologues, by mass spectrometry (Table 1). Our data are consistent with a previously unreported IR-dependent interaction between ATM and RSF1.

### Rsf1 Depletion Results in Defective DSB Repair

Depletion of the ACF1/WRCF complex, including SNF2H, has already been shown to promote sensitivity to damaging agents such as IR, MMS, and camptothecin, and mild sensitivity to UVC [5]. To assess the role of the RSF complex in the DDR, we performed clonogenic survival assays using IR and MMS treatments with single and double siRNA-dependent “knockdowns” of RSF1 and SNF2H (Figure 2D). Depletion of RSF1 results in similar IR sensitivity as depletion of SNF2H. Individual RSF1 or SNF2H depletion also resulted in sensitivity to MMS, although MMS sensitivity appeared more pronounced for the RSF1 knockdown, whereas the double knockdown appeared to have even greater MMS sensitivity, approaching that of ATM inhibition. Cells individually depleted for RSF1 and SNF2H were also mildly sensitive to the interstrand crosslinking reagent MMC (Figure S2D). RSF1-depleted cells were only weakly sensitive to UVC, whereas SNF2H-depleted cells displayed the expected mild sensitivity to UVC (Figure S2E). These data demonstrate that RSF1 functions in the cellular response to DNA damage.

**Figure 3:**
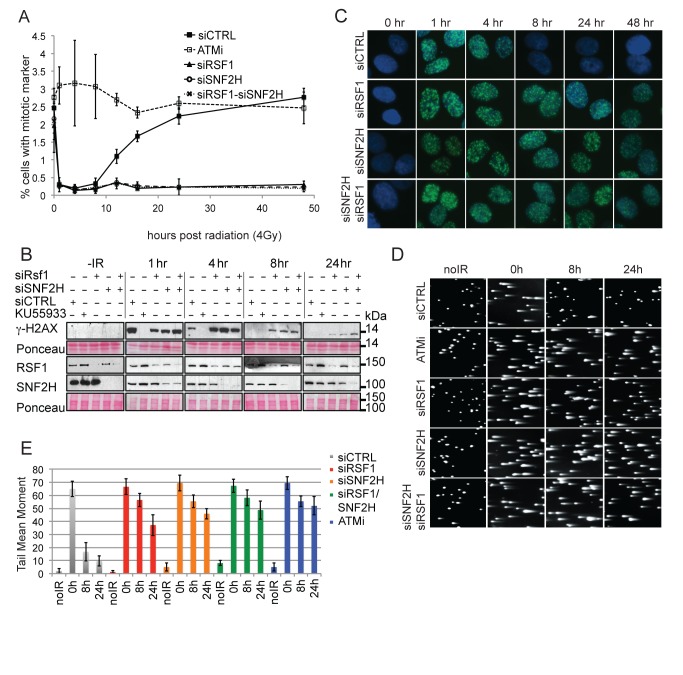
RSF1 promotes efficient DSB repair. (A) G2/M checkpoint analysis in U2OS cells. Inhibition of ATM or specific siRNA depletions are as indicated. Cells positive for the mitotic marker were followed for 48 h after irradiation. (B) Western blot analysis of γ-H2AX phosphorylation after IR (4 Gy) over 24 h in U2OS cells treated as indicated. The extent of knockdown is indicated in the RSF1 and SNF2H blots. (C) Immunofluorescence showing γ-H2AX IRIF formation and persistence in U2OS cells over 48 h postradiation (4 Gy) and treated with the indicated siRNAs. (D) Neutral comet assay showing the repair of fragmented DNA induced by 2 Gy of IR in U2OS cells treated either with an ATM inhibitor (KU55933) or with the indicated siRNAs. (E) Quantification of tail moments represented in the neutral comet assay shown in Figure 3D. Error bars indicate standard error of the mean (SEM) from three independent experiments.

In addition to its role in DSB repair [2], ATM has a well-characterized role in the G2/M checkpoint [22]. As measured by accumulation of a mitotic marker (histone H3S10 phosphorylation), single and double depletion of RSF1 and SNF2H resulted in entry into the G2/M checkpoint with normal kinetics that was similar to the scrambled siRNA control, whereas ATM-inhibited cells never entered the checkpoint (Figure 3A). However, unlike control cells, which re-entered the cell cycle upon completion of repair, RSF1- and SNF2H-depleted cells remained arrested for the duration of the experiment. Normal entry into the G2/M checkpoint followed by defective exit is consistent with either defective DSB repair or checkpoint recovery.

To distinguish between these possibilities, we monitored the γ-H2AX histone modification found at DSBs [23] by both Western blotting and immunofluorescence microscopy (Figure 3B and 3C and Figure S3A). The γ-H2AX signal was lost 4–8 h after irradiation, whereas in singly and doubly depleted RSF1 and SNF2H cells, it persisted for at least 24 h. These data indirectly indicate a DSB repair defect in the absence of RSF1 and SNF2H. We then monitored DSBs directly using neutral comet assays to confirm this result directly (Figure 3D and 3E). Comet tails, corresponding to broken DNA, that are detected immediately after IR were rapidly lost in control cells, indicative of DSB repair. These tails persist throughout the experiment in cells singly or doubly depleted for RSF1 and SNF2H. Essentially equivalent results were obtained by pulse-field gel electrophoresis (Figure S3B). Thus, both of these physical techniques confirmed defective DSB repair in RSF1- and SNF2H-depleted cells, consistent with our clonogenic survival, checkpoint, and γ-H2AX assays.

### RSF1 Is Required for the Ordered Recruitment of CENPS/MHF1–CENPX/MHF2 and FANCD2–FANCI onto Damaged Chromatin

**Figure 4:**
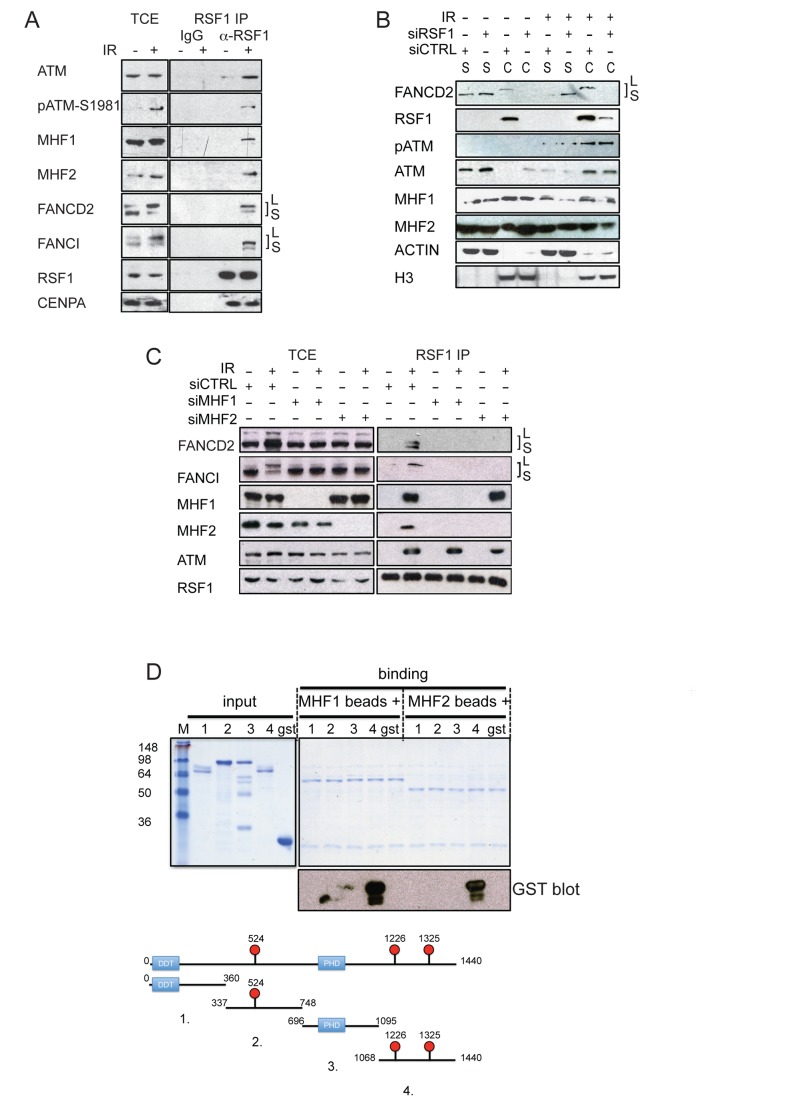
RSF1 regulates the ordered recruitment of centromeric and FA proteins onto chromatin after IR. (A) Co-immunoprecipitation of the indicated proteins with RSF1 from extracts prepared from U2OS cells that had been either mock treated or irradiated (4 Gy) and allowed to recover for 1 h before harvesting. Chromatin was solubilised by Benzonase treatment. (B) Western blot analysis of the indicated proteins after chromatin fractionation of cell extracts prepared from U2OS cells after the indicated treatments (IR was 4 Gy and siRNAs were as indicated). S and C refer to soluble and chromatin fraction, respectively. (C) Co-immunoprecipitation of the indicated proteins with RSF1 from extracts prepared from U2OS cells that had been either mock treated or irradiated (4 Gy). siRNA treatment was as indicated. (D) Five micrograms of GST-fusion proteins corresponding to RSF1 fragments (see schematic) were incubated with MBP–MHF1 beads or MBP–MHF2 beads (corresponding each to 2 µg of each recombinant protein). Elutions were blotted for GST.

Lack of RSF1 has also been reported to destabilise the centromeric histone H3 variant CENPA within centromeric DNA as it facilitates its removal by salt extraction [8]. Recruitment of CENPA to both I-Sce1–induced DSBs and laser-induced DNA damage has also been reported [24]. Although we are unable to detect CENPA localisation to IR-induced foci (IRIF) by immunofluorescence, we observed an IR- and RSF1-dependent interaction between ATM and CENPA (Figure S4A), although only after 1% PFA cross-linking. However, the interaction between RSF1 and CENPA was independent of IR (Figure 4A). Thus, we were unable to demonstrate a direct link between RSF1 and CENPA at DSBs.

As RSF1 is required for the stabilisation of CENPA into centromeric chromatin, we wondered if it could play a similar role for other centromeric proteins at DSBs. CENPS/MHF1 and CENPX/MHF2 are two additional centromeric proteins that are known to interact with FANCM and have also been localised to sites of laser-activated psoralen-induced ICLs in S-phase cells [14]. Interestingly, these two proteins form a tetrameric complex resembling the histone H3–H4 tetramer [25]. Depletion of either CENPS/MHF1 (resembling H3) or CENPX/MHF2 (resembling H4) has been shown to result in defective FANCM recruitment, defective FANCD2 and FANCI mono-ubiquitination, and defective resolution of ICLs [13],[14].

We hypothesised that the role of RSF1 in response to IR-induced DNA damage could be the recruitment of the histone fold proteins CENPS/MHF1 and CENPX/MHF2 to nucleosomes or nucleosome-like structures that occur in the vicinity of DSBs. This in turn may lead to the recruitment and mono-ubiquitination of FANCI/FANCD2 at DSBs, which in turn contribute to effective repair of breaks. In support of this hypothesis, both FANCI and FANCD2 are known ATM substrates and required for DSB resolution [10],[26]. Furthermore, our proteomic analysis of chicken Atm-interacting proteins also suggested a possible interaction with FancI (Table 1).

RSF1 co-immunoprecipitates with activated ATM (Figure 2A), and we confirmed this interaction in RSF1 immunoprecipitates (Figure 4A and 4C). Consistent with our recruitment hypothesis, RSF1 immunoprecipitates prepared from IR-treated cells contained the CENPS/MHF1 and CENPX/MHF2 proteins. We mapped the region of RSF1 that interacts with CENPS/MHF1 and CENPX/MHF2 *in vitro* using recombinant proteins (Figure 4D). Both of these histone-related proteins interacted with the C-terminus of RSF1, which contains the sites of ATM-dependent phosphorylation (Figure 2B and Figure S2G). Interestingly, the FANCD2 and FANCI proteins also interacted with RSF1 after IR (Figure 4A). Furthermore, and consistent with the RSF1 protein being exclusively bound to chromatin (Figure 2B, see also Figure 4B and Figure S4B), it was primarily the “long” forms, known to be mono-ubiquitinated and chromatin retained forms of FANCD2 and FANCI [27], that co-immunoprecipitated with RSF1 (Figure 4A and 4C).

We determined the dependency of FANCD2 and FANCI recruitment and mono-ubiquitination upon RSF1 by chromatin fractionation in the presence and absence of RSF1 (Figure 4B and Figure S4B). In un-irradiated cells that contain RSF1, the FANCD2 and FANCI proteins can be found as both soluble and chromatin retained proteins, with the mono-ubiquitinated form being largely on the chromatin. After IR, both FANCD2 and FANCI are primarily mono-ubiquitinated and retained in the chromatin fraction in cells containing RSF1. However, upon depletion of RSF1, both chromatin recruitment and mono-ubiquitination of FANCD2 and FANCI are abrogated. Importantly, expression of FLAG-tagged mouse Rsf1 rescues FANCD2 foci and mono-ubiquitination when endogenous RSF1 is depleted, confirming the specificity of our depletion conditions (Figure S4C and D). Also, using an anti-Flag monoclonal antibody, we could not localise mouse Rsf1 to IRIF, suggesting that either insufficient Rsf1 is localised to IRIFs to be detected or that the role of Rsf1 at DSBs is transient. Thus, RSF1 is required for efficient mono-ubiquitination and retention of FANCD2 and FANCI on damaged chromatin.

To address the sequence of RSF1-dependent molecular events, we repeated the RSF1 immunoprecipitation after depletion of either CENPS/MHF1 or CENPX/MHF2 (Figure 4C). In the absence of these proteins, ATM could still be detected in RSF1 immunoprecipitates, indicating that CENPS/MHF1 and CENPX/MHF2 are not required for the ATM–RSF1 interaction. However, the interaction between RSF1 and FANCD2 and FANCI could not be detected after knockdown of either CENPS/MHF1 or CENPX/MHF2. Consistent with a previous report [14], mono-ubiquitination of FANCD2 and FANCI was abrogated when either CENPS/MHF1 or CENPX/MHF2 were depleted. We also noticed that in these immunoprecipitation experiments, in the absence of CENPX/MHF2, some CENPS/MHF1 was still detected in the RSF1 immunoprecipitates but not vice versa. This suggests that the subunit of the CENPS/MHF1–CENPX/MHF2 complex that interacts directly with RSF1 is CENPS/MHF1. A direct interaction between CENPS/MHF1 and RSF1 is consistent with CENPS/MHF1 being a histone fold protein that most resembles histone H3 protein and RSF being a chromatin remodelling complex that preferentially recognises histone H3 and the H3-like CENPA [8],[25].

Thus far, our data are consistent with DSB-dependent ATM kinase activity being required for an interaction between ATM and RSF1 on chromatin; RSF1 then recruits the CENPS/MHF1–CENPX/MHF2 complex, which in turn recruits FANCD2 and FANCI. Of note, the role of RSF1 appears to be specific to DSBs, as sensitivity of RSF1-depleted cells to MMC is subtle and FAND2 foci after MMC treatment are unaffected by RSF1 knockdown (Figure S2D, H, and I).

### RSF1 and CENPS/MHF1 Are Required for Both NHEJ and HR

**Figure 5:**
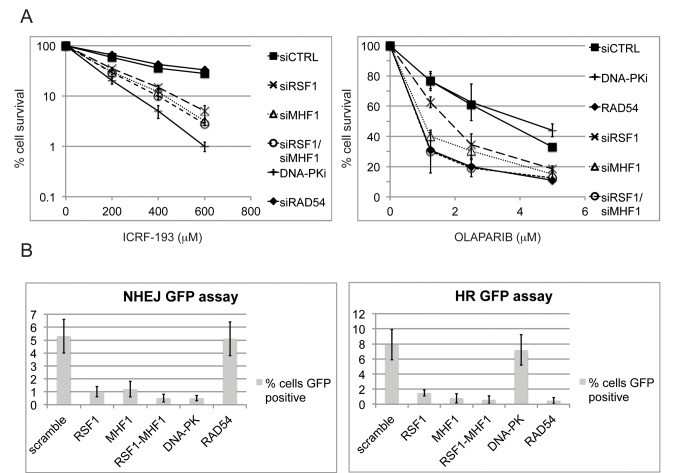
Recruitment of CENPS/MHF1–CENPX/MHF2 by RSF1 is an early event required for both NHEJ and HR. (A) Clonogenic survival after the indicated treatments of U2OS cells with ICRF-193 or Olaparib. (B) NHEJ and HR repair assays. Cell lines with stably integrated constructs specific for either NHEJ or HR were transfected with a plasmid that expresses the I-SceI endonuclease and with the indicated siRNAs. Cut sites and the EGFP gene have been transfected with a plasmid expressing the enzyme. Efficiency of reconstitution of the EGFP gene (measured by EGFP expression) is indicated. In both (A) and (B) error bars are the standard error of the mean (SEM) from three repetitions.

To determine the specificity of RSF1 for either of the two major DSB repair pathways, NHEJ and HR, we initially performed clonogenic survival assays on cells depleted of RSF1 or CENPS/MHF1 and treated with ICRF-193 or Olaparib (Figure 5A). ICRF-193 is an inhibitor of topoisomerase 2 and generates DSBs that are repaired specifically by NHEJ [28],[29], whereas Olaparib is a PARP inhibitor that prevents repair of single-strand breaks, resulting in DSBs in the S phase that are normally repaired by HR [30],[31]. Both of these drugs resulted in sensitivity in both RSF1- and CENPS/MHF1-depleted cells. In order to have a more direct and robust quantification of the NHEJ and HR pathways, we utilised established NHEJ and HR assays that are based upon expression of a GFP reporter after induction of a site-specific DSB by the *I-SceI* endonuclease [32],[33]. Depletion of RSF1 and/or CENPS/MHF1 resulted in impaired repair in both of these assays (Figure 5B). Intriguingly, we also noticed defective recruitment of CtIP to IRIF upon depletion of RSF1 (Figure S5C). These data are consistent with RSF1-dependent recruitment of CENPS/MHF1 being required for efficient DSB repair by both major DSB repair pathways.

### RSF1 Is Required for Recruitment of CENPS/MHF1–CENPX/MHF2 and FANCD2–FANCI into IRIF

**Figure 6:**
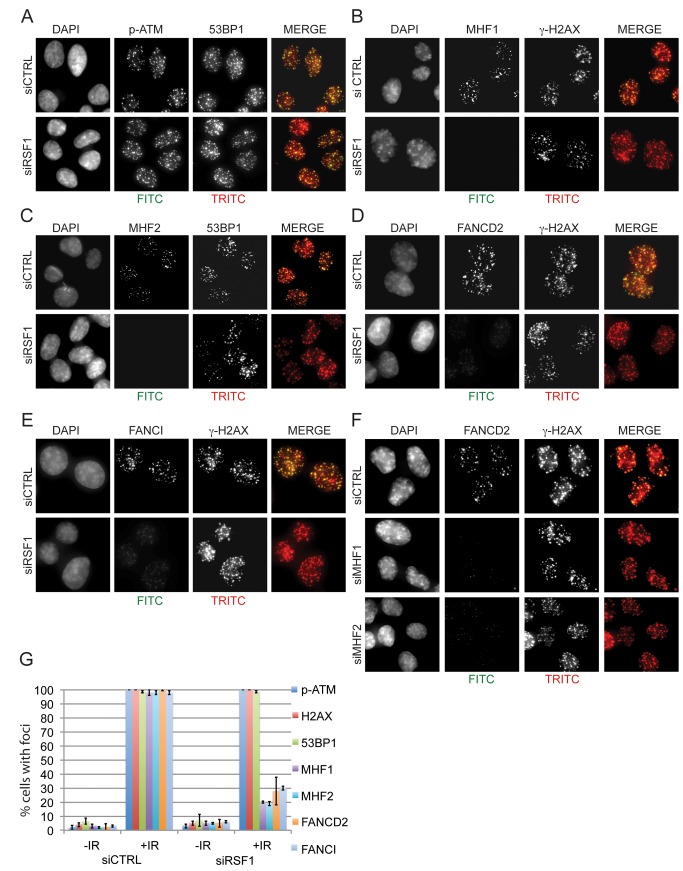
RSF1 is required for the recruitment of CENPS/MHF1, CENPX/MHF2, FANCD2, and FANCI to IRIF. (A–F) Immunofluorescence of the indicated proteins 60 min after IR (4 Gy) in the indicated siRNA-treated U2OS cells: (A) p-ATM and 53BP1, (B) MHF1 and γ-H2AX, (C) MHF2 and 53BP1, (D) FANCD2 and γ-H2AX, (E) FANCI and γ-H2AX, and (F) FANCD2 and γ-H2AX. (C) Quantification of pATM, γ-H2AX, 53BP1, MHF1, MHF2, FANCI, and FANCD2 IRIF (when cells were depleted of RSF1). At least 100 cells were counted for each set of data; cells with more than 10 foci were considered positive. Error indicates SEM.

Previously, the recruitment of FANCM and efficient mono-ubiquitination of FANCD2 after induction of DNA ICLs was shown to be dependent upon MHF1 [14]. We therefore investigated IR-dependent focal recruitment of CENPS/MHF1, CENPX/MHF2, FANCD2, and FANCI after knockdown of RSF1 (Figure 6). Note that our extraction protocol distinguishes between the more dynamic recruitment of CENPS/MHF1 and CENPX/MHF2 to centromeres relative to their more stable recruitment to IRIFs (Figure S6A to D). Although RSF1-depleted cells are proficient for recruitment of ATM and 53BP1 (Figure 6A) into IRIF, as well as for γ-H2AX (Figure 6B) IRIF, they are deficient for CENPS/MHF1 (Figure 6B) and CENPX/MHF2 (Figure 6C) IRIF. As expected, this then results in loss of FANCD2 (Figure 6D) and FANCI (Figure 6E) IRIF. Quantification of cells with at least 10 foci revealed that although ATM, γ-H2AX, or 53BP1 IRIF were unaffected by RSF1 depletion, CENPS/MHF1, CENPX/MHF2, FANCD2, and FANCI foci were reduced 3–4-fold relative to the undepleted control cells (Figure 6G).

The recruitment of CENPS/MHF1 and CENPX/MHF2 into IRIF contrasts with our failure to observe CENPA in IRIF, despite the recruitment of CENPA to kinetochores being readily apparent although clearly distinct from CENPS/MHF1 or NBS1 at IRIF (Figure S6G). In fact, depletion of CENPS/MHF1 does not disrupt kinetochore formation as assessed by CENPA recruitment, nor did it perturb mitotic arrest and release after nocodazole addition and removal (Figure S6H), suggesting that the residual levels of CENPS/MHF1 and CENPX/MHF2 after knockdown are sufficient for their function at kinetochores. We also confirmed that CENPS/MHF1 and FANCI IRIF are mainly co-localised (Figure S6E). Next, we determined the dependence of FANCD2 and FANCI foci on CENPS/MHF1 and CENPX/MHF2. Depletion of CENPS/MHF1 or CENPX/MHF2 results in loss of FANCD2 IRIF (Figure 6F) and FANCI (Figure S6F). Thus, our immunofluorescence data are in agreement with our biochemical data (Figure 4C) and together suggest a hierarchical recruitment of these proteins to DSBs.

Together, our data suggest a novel role for RSF1 in DSB repair in which this chromatin remodelling activity is required for recruitment of the CENPS/MHF1–CENPX/MHF2 histone fold proteins to the vicinity of IR-induced DNA lesions. These proteins are in turn required for both FANCD2–FANCI recruitment and their efficient mono-ubiquitination.

## Discussion

ATM is the apical regulator of a complex cascade of molecular events required for repair of DNA DSBs trigger [2],[34]. Using a quantitative proteomics strategy, we report a stable interaction between ATM and the chromatin-remodelling factor RSF1. Previously, RSF1, which together with SNF2H, an ATP-dependent DNA helicase, forms the RSF complex, has been shown to be required for nucleosome deposition and regularly spaced nucleosome arrays throughout the genome [7],[35]. Additionally, it has been shown to be required for correct assembly of the CENPA complex at centromeres [8].

### RSF1 Contributes to DSB Repair

The ATM–RSF1 interaction is dependent upon IR and has been observed in both chicken DT40 cells and human cell lines. Importantly, this interaction is also dependent upon the kinase activity of the ATM kinase, which is consistent with the previous identification of RSF1 as a substrate of the ATM kinase [10]. Using GST–ATM fusions [36] we have mapped the region of ATM that interacts with RSF1 to the C-terminal FATC domain, a region also reported to interact with TRF1 in this assay [37]. RSF1 depletion results in sensitivity to IR and MMS but only moderate sensitivity to MMC and UVC. A role in DSB repair was suggested by persistence of the γ-H2AX modification and the failure of RSF1-depleted cells to exit the G2/M checkpoint. We confirmed defective DSB repair using two physical techniques, the comet assay and pulse-field gel electrophoresis. Moreover, using assays specific to either direct end joining or homology-directed repair of induced DSBs, our results indicate that RSF1 is required for efficient DNA repair by either of these two mechanisms. Together, our data indicate that RSF1 is a new player in the DDR required for the efficient repair of DSBs.

### RSF1 Is Required for the Ordered Recruitment of Centromeric and FA Factors onto Damaged Chromatin

Recent reports have shown that after DNA ICLs, two proteins, FANCM-interacting histone fold proteins 1 and 2 (MHF1 and MHF2), are recruited to these lesions, resulting in the stabilisation of FANCM and the subsequent recruitment and mono-ubiquitination of FANCD2 and FANCI [13],[14]. Previously, these proteins had been implicated in centromere function and termed CENPS and CENPX, respectively [38]. Structurally, CENPS/MHF1 and CENPX/MHF2 form a tetramer that resembles the histone H3–H4 tetramer [25].

RSF1 is a chromatin-remodelling factor with ATPase activity required for the establishment of centromeric CENPA, which in turn is required for the subsequent localisation of CENPS/MHF1 and CENPX/MHF2 to centromeres [8],[38]. This role led us to determine whether RSF1 might be required for the recruitment of CENPS/MHF1 and CENPX/MHF2 to DSBs. RSF1 appears to specifically interact with CENPS/MHF1 as loss of CENPX/MHF2 does not abolish the RSF1 interaction with CENPS/MHF1, whereas loss of CENPS/MHF1 does abolish the interaction. This is also consistent with crystallographic studies indicating that although CENPS/MHF1 is not a histone variant, it is a histone fold protein that most closely resembles histone H3 and RSF1 is known to preferentially bind to histone H3 and CENPA [7],[8]. Using GST–RSF1 fusion proteins and recombinant CENPS/MHF1 and CENPX/MHF2, we have also demonstrated a direct interaction between RSF1 and these histone fold proteins. CENPS/MHF1 and CENPX/MHF2 interact with the C-terminus of RSF1, a region containing sites of *in vivo* ATM-dependent phosphorylation.

Importantly, using depletion of CENPS/MHF1 and CENPX/MHF2, we have also shown that these proteins are subsequently required for the recruitment and mono-ubiquitination of FANCD2 and FANCI at DSBs. Note that previous reports have also demonstrated FANCD2 and FANCI localization to DSBs produced during the S phase [17],[27]. Although there are connections between the FA pathway and replication-coupled, homology-directed DSB repair [16],[18], the precise role of activated FANCD2 and FANCI in DSB repair has not yet been characterised. However, it is likely that coordinated ubiquitination, which regulates recruitment of specific factors to the mono-ubiquitin moiety via a plethora of ubiquitin binding domains (UBDs), and de-ubiquitination of FANCD2 and FANCI are required for efficient repair.

A role for centromeric proteins in DSB repair has also been suggested by the reported localisation of CENPA to laser-induced and I-SceI–induced site-specific DSBs [24]. Unfortunately, under our experimental conditions we could not detect CENPA localisation to sites of IRIF, although its localisation to kinetochores was readily apparent. Nor could we detect an interaction between ATM and CENPA by either mass spectrometric analysis or by co-immunoprecipitation. However, we could detect RSF1, CENPS/MHF1, and CENPA in ATM immunoprecipitates prepared from cells treated with formaldehyde, an agent that crosslinks proteins that are in close proximity. Our data do not rule out a possible role for CENPA at DSBs. However, future studies will be required to determine the precise role of CENPA, if any, in DSB repair.

**Figure 7:**
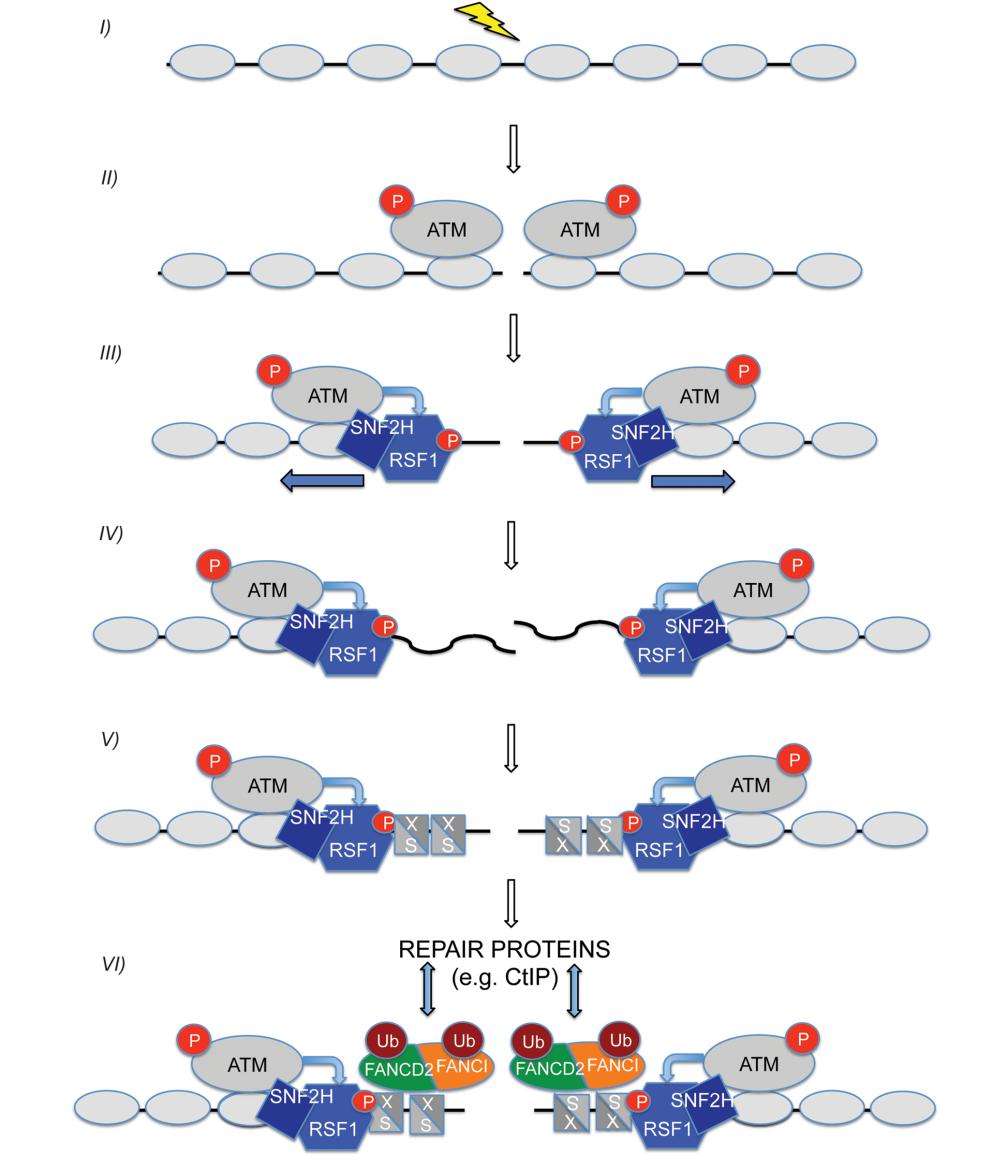
ATM-dependent activation of the RSF complex facilitates DNA DSB repair. Upon damage (step I) ATM is activated and recruited to chromatin containing a DSB (step II). One of the substrates of ATM is the RSF1 protein. Once activated by ATM-dependent phosphorylation of RSF1 (step III), we propose that the RSF chromatin-remodelling complex facilitates DSB repair utilising its translocase activity, via an ill-understood mechanism likely to involve nucleosome sliding (step III) and even nucleosome clearance (step IV). As part of this process, CENPS/MHF1 and CENPX/MHF2, two histone-fold proteins, are recruited to the vicinity of the DSB in an RSF-dependent fashion (step V). As the CENPS/MHF1–CENPX/MHF2 tetramer has fewer contacts with DNA than a nucleosome or the H3–H4 tetramer [25], it could perform a “*placeholder*” function, preventing DNA that has been unwound from nucleosomes on either side of the DSB from forming a random coil and also facilitating the subsequent access of DSB repair machineries. Downstream of CENPS/MHF1 and CENPX/MHF2 recruitment, the FANCD2 and FANCI proteins are recruited and mono-ubiquitinated by a mechanism dependent upon prior recruitment of CENPS/MHF1 and CENPX/MHF2 (step 6). It is not know how many steps exist between CENPS/MHF1–CENPX/MHF2 and subsequent FANCD2–FANCI recruitment. Mono-ubiquitinated FANCD2 and FANCI presumably functions as docking sites that regulate recruitment of subsequent DSB repair proteins, such as CtIP, which has functions in both NHEJ and HR.

In summary, we have identified RSF1 as a new player in the DDR response that is required for repair of DSBs. Our data are consistent with a model (Figure 7) in which DSB-activated ATM results in RSF-dependent recruitment of the CENPS/MHF1–CENPX/MHF2 complex, which in turn is required to recruit and activate FANCD2 and FANCI. Although this sequence of events is not required for DSB-dependent checkpoint signalling, it is required for DSB repair, probably in constructing a chromatin environment permissive for repair. This is likely to be a highly dynamic process requiring transient complexes between DNA and CENPS/MHF1–CENPX/MHF2 complexes.

## Materials and Methods

### Growth and Transfection of DT40 Cells

Cells were grown at 39°C to a maximum density of 1×10^6^ cells/ml in RPMI (Lonza) supplemented with 10% foetal calf serum (Lonza), 1% chicken serum (Lonza), and 1% penstrep (Lonza). For transfection, 1×10^6^ cells were collected and resuspended into 0.5 ml PBS and transferred to transfection cuvettes (Bio-rad catalog number 165–2088, 0.4 cm electrode gap), and 5–15 µg of linearised DNA was added. After an incubation (10 min/RT), electroporation was performed using a gene pulsar apparatus (Bio-rad, 550 V, 25 µF). Cells were then grown for two doubling times (approximately 16–24 h). The media was replaced with fresh RPMI media containing the required drug for selection, and the cells were aliquoted into 4× 96-well plates. When the clones grew big enough to be visible, they were transferred into 24-well plates (containing 1 ml of media in each well). Upon confluence they were split into 12-well dishes (4 ml in total), and once confluent, 2 ml was harvested and frozen at −80°C in freezing media (serum plus 10% DMSO) and 2 ml (∼1.5×10^6^ cells) harvested for genomic DNA extraction.

### siRNA in Human Cells

We transfected 20 nmol of siRNA (Dharmacon) per 35 mm tissue culture dish of cells (Oligofectamine, Invitrogen) onto cells at 70% confluence according to the manufacturer's instructions. After 48 h, the siRNA transfection was repeated and the cells were harvested the next day. For siRNA sequences, see Table 2.

**Table 2:** siRNA sequences used in this work.

Pool Catalog Number	Gene Symbol	Gene Accession	Sequence
MU-020374-00	RSF1	NM_016578	GGAAAGACAUCUCUACUAU
MU-020374-00	RSF1	NM_016578	UAAAUGAUCUGGACAGUGA
MU-020374-00	RSF1	NM_016578	GAACAGAGUCCUUAAAUUC
MU-020374-00	RSF1	NM_016578	GGACUUACCUUCAACCAAU
MU-003202-05	ATR	NM_001184	GAACAACACUGCUGGUUUG
MU-003202-05	ATR	NM_001184	GCAACUCGCCUAACAGAUA
MU-003202-05	ATR	NM_001184	UCUCAGAAGUCAACCGAUU
MU-003202-05	ATR	NM_001184	GAAUUGUGUUGCAGAGCUU
MU-011478-00	SMARCA5	NM_003601	GGAUUAAACUGGCUCAUUU
MU-011478-00	SMARCA5	NM_003601	GAGGAGAUGUAAUACCUUA
MU-011478-00	SMARCA5	NM_003601	GGAAUGGUAUACUCGGAUA
MU-011478-00	SMARCA5	NM_003601	GGGCAAAUAGAUUCGAGUA
M-032895-01-0005	MHF1	NM_199294	UCGCCCGGCCACCUCCGCA
M-032895-01-0005	MHF1	NM_199294	GAUUCUCUUACCAACAGAG
M-032895-01-0005	MHF1	NM_199294	GAUUAACCUAGAACGAAAA
M-032895-01-0005	MHF1	NM_199294	GUAAUUAAAAUUGUGUGAG
M-016829-01-0005	MHF2	NM_144998	GCGCGUUGAGGCUGCGGUC
M-016829-01-0005	MHF2	NM_144998	GAAGGAUUUGGAGUUGAUU
M-016829-01-0005	MHF2	NM_144998	GGCAGGACUGCAGUGCACU
M-016829-01-0005	MHF2	NM_144998	GCAGGUAGCCUGUGUGUUU

### DT40 Genomic DNA Extraction and Southern Blotting

The 1.5×10^6^ cells were collected and resuspended in 500 µl TAIL buffer (Tris-HCl pH 8.8, 50 mM, EDTA pH 8 100 mM, NaCl 100 mM, SDS 1%, 3 µl of a 20 mg/ml Proteinase K solution for every 0.5 ml TAIL buffer) in Eppendorf tubes, and the cells were incubated overnight at 37°C. The Eppendorf tubes were shaken vigorously for 5 min, 200 µl of saturated NaCl (6 M) was added to each, and the tubes were shaken vigorously for a further 5 min. Debris was removed by centrifugation (14,000 rpm/30 min at 4°C), and 700 µl of ice-cold isopropanol was added to each supernatant and mixed by gentle inversion of the tube. Genomic DNA was pelleted by centrifugation (14,000 rpm/10 min) and washed with 300 µl of 70% ice-cold ethanol. The genomic DNA pellet was briefly air dried and then resuspended in 60 µl TE buffer at pH 7.5–8.0, plus 2 µl of a 5 mg/ml RNAse A. Southern blotting was performed according to the manufacturer's (DIG-PCR-DNA labelling and probing, Roche) instructions

### Floxing in DT40 Cells with pANMerCreMer and 4-OH-Tamoxifen

The 5×10^6^ cells were harvested and resuspended in 100 µl of Nucleofector solution (Amaxa), to which 75 µg of endotoxin-free pANMerCreMer plasmid DNA was added. The mix has been transferred to an Amaxa cuvette for “nucleofection.” Nucleofected cells were added to 10 ml of RPMI containing 100 nM 4-OH-Tamoxifen and after growth for 24 h plated into 96-well plates at 0.5/1/1.5 cells per plate. After 7–8 d, subclones were picked and replated under selection for further analyses.

### Purification of ATM in DT40 Cells

A total of 1 l of WT and HFSC-ATM cells were grown in petri dishes to a maximum confluency of 1×10^6^ cells/ml, harvested, resuspended in 0.5 l of the conditioned media, and irradiated with 10 Gy. After 1 h recovery at 39°C, they were washed (1× PBS; ice-cold) and resuspended in 2 ml Lysis Buffer [50 mM Tris-HCl, pH 7.5; 150 mM NaCl; 0.5% NP-40; 1× Protease inhibitors (Roche tablet); 1× phosphatase inhibitors (SIGMA)] at 4°C for 30 min. Chromatin was solubilised by adding MgCl_2_ (to a final concentration of 1 mM) and Benzonase (250 unit/ml, Sigma) and undergoing incubation for 45 min at 4°C. The reaction was stopped by addition of EDTA (to a final concentration of 1 mM) and incubation on ice for 5 min. Debris was pelleted (100,000 g/60 min/1 h) and the supernatant harvested and quantified by Bradford assay. Precisely the same amount of total cell lysates for the two samples were mixed ensuring a 1∶1 ratio and purified using a Gravity Flow Strep-tagII column (Iba Tagnology), following the manufacturer's instructions. The eluted fraction containing the protein was lyophilised and sent on dry ice to Dundee Cell Products (Scotland) for mass spectromeric analysis.

### Preparation of Samples for MS Analysis

Equal amounts of protein from unlabelled and labelled samples were combined prior to protein digestion. Briefly, samples were reduced in 10 mM DTT and alkylated in 50 mM Iodoacetamide prior to boiling in loading buffer, and then separated by 1D SDS-PAGE (4%–12% Bis-Tris Novex mini-gel, Invitrogen) and visualized by colloidal Coomassie staining (Novex, Invitrogen). The entire protein gel lanes were excised and cut into 10 slices each. Every gel slice was subjected to in-gel digestion with trypsin overnight at 37°C. The resulting tryptic peptides were extracted by formic acid (1%) and acetonitrile, lyophilized in a speed-vac, and resuspended in 1% formic acid.

### Mass Spectrometry

Trypsin-digested peptides were separated using an Ultimate 3000 RSLC (Thermo Scientific) nano-flow LC system. On average 0.5 µg was loaded with a constant flow of 5 µl/min onto an Acclaim PepMap100 nano-Viper C18 trap column (100 µm inner-diameter, 2 cm; Thermo Scientific). After trap enrichment, peptides were eluted onto an Acclaim PepMap RSLC nano-Viper, C18 column (75 µm, 15 cm; ThermoScientific) with a linear gradient of 2%–40% solvent B (80% acetonitrile with 0.08% formic acid) over 65 min with a constant flow of 300 nl/min. The HPLC system was coupled to a linear ion trap Orbitrap hybrid mass spectrometer (LTQ-Orbitrap Velos, Thermo Scientific) via a nano-electrospray ion source (Thermo Scientific). The spray voltage was set to 1.2 kV, and the temperature of the heated capillary was set to 250°C. Full-scan MS survey spectra (*m*/*z* 335–1800) in profile mode were acquired in the Orbitrap with a resolution of 60,000 after accumulation of 1,000,000 ions. The 15 most intense peptide ions from the preview scan in the Orbitrap were fragmented by collision-induced dissociation (normalized collision energy, 35%; activation Q, 0.250; and activation time, 10 ms) in the LTQ after the accumulation of 10,000 ions. Maximal filling times were 1,000 ms for the full scans and 150 ms for the MS/MS scans. Precursor ion charge state screening was enabled, and all unassigned charge states as well as singly charged species were rejected. The lock mass option was enabled for survey scans to improve mass accuracy [39]. Data were acquired using the Xcalibur software.

### Quantification and Bioinformatics Analysis

The raw mass spectrometric data files obtained for each experiment were collated into a single quantitated data set using MaxQuant (version 1.2.2.5) [40] and the Andromeda search engine software [41]. Enzyme specificity was set to that of trypsin, allowing for cleavage N-terminal to proline residues and between aspartic acid and proline residues. Other parameters used were as follows: (i) variable modifications, methionine oxidation, protein N-acetylation, gln → pyro-glu; (ii) fixed modifications, cysteine carbamidomethylation; (iii) database, target-decoy human MaxQuant (ipi.HUMAN.v3.68); (iv) heavy labels, R6K4 and R10K8; (v) MS/MS tolerance, FTMS, 10 ppm; ITMS, 0.6 Da; (vi) maximum peptide length, 6; (vii) maximum missed cleavages, 2; (viii) maximum of labelled amino acids, 3; and (ix) false discovery rate, 1%. Peptide ratios were calculated for each arginine- and/or lysine-containing peptide as the peak area of labelled arginine/lysine divided by the peak area of nonlabelled arginine/lysine for each single-scan mass spectrum. Peptide ratios for all arginine- and lysine-containing peptides sequenced for each protein were averaged.

### Immunoprecipitation in Human Cells

U2OS or HEK293 cells were grown to 80%–90% confluency in a single 10 cm petri dish. Cells were trypsinized and harvested, the pellet was washed in 1×PBS (ice-cold), and resuspended in 0.5–1 ml of lysis buffer [50 mM Tris, pH 7.5; 150 mM NaCl; 0.5% NP-40; 5% glycerol; 1× Protease inhibitors (Roche tablet); 1× phosphatase inhibitors (Sigma)] supplemented with 1 µl per 1 mL Benzonase (250 units/ml, Sigma) to solubilize chromatin and incubated at 4°C for 45 min. After incubation, lysates were cleared by centrifugation for 30 min at 4°C, 14,000 rpm, and quantified by Bradford assay. For salt extraction of chromatin bound proteins, cell pellets were resuspended in 200 µl of lysis buffer containing 400 mM NaCl and incubated at 4°C for 20 min. Additional 400 µl of lysis buffer (without salt) were added, and lysates were cleared by centrifugation (14,000 rpm, 30 min, 4°C). For immunoprecipitation, equal amounts of total protein extracts (typically 10 mg) were used for each sample and typically 1 µg of primary antibody per mg of extract was added and left in ice for 2 h. We added 40 µL pre-equilibrated protein G beads (GE Healthcare) and left it at 4°C with gentle agitation for a further 2 h. The beads were gently pelleted (1,000 rpm/5 min/4°C), washed six times with 1× lysis buffer, and resuspended in 50 µL of sample loading buffer (Invitrogen).

### Pulldown Assays

Pulldown assays were performed according to [37]. Briefly GST–ATM fusion plasmids [36], GST–RSF1 fragments, and MBP–MHF1/2 were transformed into Rosetta cells. A 20 ml overnight culture was used to inoculate 1 l of LB-Amp (50 µg/ml)–Chloramphenicol (50 µg/ml), and at OD600, 0.5–0.7, 0.4 mM IPTG (final) was added and incubated overnight at 20°C. Cells were harvested, resuspended in 30 ml of lysis buffer (50 mM Tris [pH 7.9], 100 mM KCl, 1% Triton X-100, 2 mM DTT, 0.1 mM PMSF, complete protease inhibitor tablet [Roche]), and sonicated three times for 30 s on ice. The lysate was cleared by centrifugation at 50,000 *g* at 4°C and incubated with 200 µl of equilibrated glutathione beads for 2 h at 4°C. The beads were washed three times for 10 min each (washes 1 and 3, PBS, 1% Triton X-100, 2 mM DTT, 0.1 mM PMSF, 1 mM benzamidine, 1 complete protease inhibitor tablet [Roche]; wash 2, 300 mM NaCl, 50 mM Tris [pH 7.9], 2 mM DTT, 0.1 mM PMSF, 1 mM benzamidine, 1 complete protease inhibitor tablet [Roche]) and a fourth time in wash 4 (50 mM Tris [pH 7.9], 100 mM KCl, 50% glycerol, 2 mM DTT, 0.1 mM PMSF).

### *In Vitro* Binding Assay

GST–RSF1 fusion proteins were eluted in 500 µl of wash 4 (see pulldown assay) containing 15 mM glutathione (reduced form). Five micrograms of GST fusion proteins or GST alone were incubated with 2 µg of MBP–MHF1 or MBP–MHF2 beads in binding buffer (150 mM NaCl, 100 mM KCl, 50 mM Tris [pH 8.0], 1% NP40, 0.1% SDS, 100 µg/ml BSA) at 4°C for 1 h.

Beads were collected by centrifugation at 5,000 rpm at 4°C and washed three times for 10 min each with binding buffer, and bound protein was eluted by boiling the samples in Laemmli buffer. GST–RSF1 fusion proteins were detected by immunoblotting.

### Chromatin Fractionation

Cell extracts were prepared as for immunoprecipitations, except benzonase was not added during cell lysis and the cells were resuspended in 0.1 ml of lysis buffer. The supernatants were collected as soluble (“S”) fractions and the pellets (“C”) resuspended in 0.1 ml of lysis buffer supplemented with 1 µl per 1 ml Benzonase (250 units/mL, Sigma), together with 1 mM (final concentration) of MgCl_2_, to solubilize chromatin and incubated at 4°C for 45 min. Lysates were cleared (14,000 rpm, 30 min, 4°C) and quantified by Bradford assay.

### G2/M Checkpoint Analysis

Mock and treated cells (2×10^6^ cells) were harvested, resuspended in 1 ml 1× PBS, fixed by adding 3 mL of 70% ethanol, and the cells stored at −20°C. The cells were pelleted; washed in PBS; resuspended in 50 µl of PBS containing 0.25% Triton X-100, 1% BSA, and 1 µl phosphoH3-S10 antibody (Millipore); and incubated for 2 h at RT. Cells were washed once more; resuspended in 50 µl PBS containing 0.25% Triton X-100, 1% BSA, and goat anti-rabbit IgG FITC conjugate (1∶50 dilution, Jackson ImmunoResearch); and incubated (1 h/RT) in the dark. Cells were then resuspended in 0.3 ml of PBS containing propidium iodide at 25 µg/ml and RNaseA at 250 µg/ml and incubated (30 min/RT) in the dark. Cells positive for phospho-H3S10 were quantified on a FACSCanto (BD Biosystem) using FACSdiva software (BD Biosystem).

### Immunofluorescence

Cell were grown on poly-D-lysine coverslips, removed from culture, and permeabilised in 100 mM NaCl, 300 mM sucrose, 3 mM MgCl_2_, 10 mM Pipes, pH 7.5, 0.4% Triton-X for 10 min at 4°C. Cells were then fixed with 4% paraformaldehyde (PFA) for 15 min at room temperature and washed once with 1× TBS. After blocking for an hour at 37°C with 1% BSA, the primary antibody was added at an appropriate concentration in 1% BSA and incubated at 37°C for 1 h (anti–pATM-S1981 was used at 1∶200, anti–γ-H2AX was used at 1∶1,000, all antibodies from Bethyl laboratories were used at 1∶50, anti-FANCD2 antibody at 1∶20, and anti-MHF1 and anti-MHF2 [14] at 1∶200). The appropriate secondary antibody (either FITC or TRITC conjugated) was used at 1∶400 in 1× TBS at 37°C for 1 h in the dark. Images were acquired using a wide field Olympus Biosystem microscope and the Velocity software.

### Antibodies

The antibody against chicken Atm was raised by Pocono Rabbit Farm (Canada); ATM human antibody, SNF2H, BAZ1A, RIF1, and FANCI were all purchased from Bethyl Laboratories; and the RSF1 monoclonal antibody, phospho-ATM-S1981, and γ-H2AX were from Millipore and NBS1 antibody from Novus. Human ATR and FANCD2 antibody were from Santa Cruz. Histone H3.1, Actin, and BAZ1B were purchased by Abcam. CENPA antibody was a kind gift from Dr. Kevin Sullivan. MHF1 and MHF2 were a kind gift from Dr. Weidong Wang (Baltimore, Maryland), and the FANCD2 antibody was a kind gift from Professor Minoru Takata (Kyoto, Japan).

### Clonogenic Survival Assay Using DT40 Cells

Methyl cellulose medium was poured into 10 cm tri-section dishes (Iwaki Cell Biology), and cells were plated in triplicate as follows: for 0 and 2 Gy, 50 cells were plated; for 4 Gy, 500 cells were plated; and for 10 Gy, 5,000 cells were plated. After irradiation, cells were incubated at 39°C for 7–9 d and the number of colonies was counted.

### Clonogenic Survival Assay Using U2OS Cells

After either siRNA (Dharmacon) or ATM inhibitor (KU55933, Selleckchem; added 1 h before irradiation) treatment at the concentration recommended by the respective supplier, cells were counted and 500 cells were plated in 6 cm dishes for each dose used. After irradiation, cells were incubated at 37°C for 8–10 d, the media was removed, and the colonies visualized by staining with 0.25% dimethyl methylene blue (Sigma) and 50% ethanol for 45 min at room temperature. In the case of ICRF-193 and Olaparib treatment, drugs were added directly to the media and the cells were incubated for 8–10 d. Media was then discarded, and colonies were stained as indicated above.

### Pulse Field Gel Electrophoresis

We modified a published [42] protocol. Briefly, 1×10^6^ cells were harvested, resuspended in 50 µl ice-cold PBS, and mixed with an equal volume of 1% LMP agarose (Sigma). The mixture of agarose and cells was poured in a casting mold (Bio-rad) and allowed to solidify at room temperature. The plugs containing the cells were extruded into 3 ml of Lysis Buffer (10 mM Tris-HCL, pH 7.5, 50 mM EDTA, 1% Sarcosyl, and 2 mg/ml Proteinase K) and incubated for 48 h at 50°C. An 0.8% InCert agarose (BioRad) gel was cast around the plugs, and the gel was run for 30 h at 14°C, 4 V/cm with a switch time every 300 s.

### Neutral Comet Assay

Neutral Comet Assay was performed according to the manufacturer's (Neutral Comet Assay, Trevigen) protocol.

### NHEJ and HR Assays

NHEJ assays were performed as previously described in [32], and HR assays were performed as described in [33].

## Supporting Information

Figure S1 The HFSC-tag. (A) Schematic of HFSC–Atm and amino acid sequence of the HFSC-tag that encodes four tandem affinity purification epitopes (HA, Flag, Strep-tag II, and calmodulin binding protein) and a 19 amino acid linker to insulate the tag from the N-terminus of the tagged protein.
(TIF)

Figure S2 Analyses of ATM-interacting proteins using HEK293 cells and cell survival after MMC and UVC treatments. (A and B) Co-immunoprecipitation, using extracts prepared from HEK293 cells, of the indicated proteins with ATM. Chromatin bound proteins were solubilised by either benzonase (A) or, alternatively, 450 mM NaCl (B) treatments (see Materials and Methods) during cell lysis. HEK293 cells were either mock treated or treated with 10 Gy IR and harvested 1 h after irradiation. Where indicated the ATM inhibitor KU55933 was added directly to the media an hour before irradiation. (C) Co-immunoprecipitation from Figure 2A confirms, additionally, interaction of BAZ1A and BAZ1B proteins with ATM, using extracts prepared from U2OS cells. Blots of total ATM, pATM, NBS1, and HP1β are the same as in Figure 2A. Chromatin bound proteins were solubilised by benzonase treatment during cell lysis (see Materials and Methods). U2OS cells were either mock treated or treated with 10 Gy IR and harvested 1 h after irradiation. Where indicated the ATM inhibitor KU55933 was added directly to the media an hour before irradiation. (D and E) Survival of U2OS cells after treatment with MMC (D) or UVC (E) at the indicated doses and siRNA or ATMi (KU55933) treatments. Error bars indicate standard error of the mean (SEM) from three independent experiments. (F) Western blot analysis of the indicated proteins after chromatin fractionation of cell extracts prepared from U2OS cells. Note that in this assay, benzonase was added to the material pelleted after cell lysis (see Materials and Methods). (G) RSF1 immunoprecipitation. Cells were mock treated, treated with 5 Gy of IR and 5 Gy of IR plus ATM inhibitor, and harvested 1 h post-IR. Elutions were blotted with the RSF1 monoclonal antibody (Millipore) and anti–BRCA1-pS1423. The schematic to the right shows an alignment of BRCA1-S1423 with the three consensus PIK kinase sites of RSF1. (H) Immunofluorescence of the FANCD2 and γ-H2AX proteins after 24 h incubation with 1 µM MMC in the indicated siRNA-treated U2OS cells. (I) Western blot showing efficiency of FANCD2 mono-ubiquitination after 24 h incubation with 1 µM MMC in cells depleted for RSF1 or ATR as indicated.
(TIF)

Figure S3 Quantification of γH2AX foci and pulse-field gel analysis of DSB repair. (A) Quantification of γH2AX IRIF cells represented in Figure 2C. Cells with greater than 10 γH2AX IRIF were scored as positive. Error bars indicate standard error of the mean (SEM) from three independent experiments. (B) Analysis of fragmented DNA after IR (10 Gy) by pulse-field gel electrophoresis. Time postirradiation is indicated in hours. Also indicated are the respective siRNA or ATMi (KU55933) treatments. The asterisk indicates the fragmented DNA detected under the electrophoretic conditions used.
(TIF)

Figure S4 The RSF complex promotes DSB repair and interacts with centromeric proteins. (A) Co-immunoprecipitation of the indicated proteins from U2OS cells with ATM from the soluble and chromatin fraction. Note that the chromatin was solubilized by benzonase treatment. The cells were cross-linked (1% PFA treatment for 10 min at room temperature) prior to harvesting and were either mock treated or irradiated (10 Gy). (B) Western blot analysis of the indicated proteins after chromatin fractionation of cell extracts prepared from U2OS cells after the indicated treatments (IR was 4 Gy and siRNAs were as indicated). S and C refer to soluble and chromatin fraction, respectively. (C) Immunofluorescence of FANCD2 and γ-H2AX 1 h after IR (4 Gy) in the indicated siRNA-treated U2OS cells. Formation of FANCD2 IRIF is rescued by expression of Flag-tagged mouse Rsf1 in cells in which endogenous human RSF1 has been depleted. (D) Western blotting showing successful expression of Flag-tagged mouse Rsf1 in U2OS cells. Cells depleted of endogenous human RSF1 expressing Flag-mRsf1 display normal levels of mono-ubiquitination of FANCD2.
(TIF)

Figure S5 Efficiency of RSF1, CENPS/MHF1, and RAD54 depletion. (A and B) Typical knockdown efficiency of siRNA used for NHEJ (A) and HR (B) assays. (C) Immunofluorescence of CtIP and γ-H2AX 3 h after IR (4 Gy) in the indicated siRNA-treated U2OS cells.
(TIF)

Figure S6 RSF1 regulates FANCI through the centromeric protein MHF1. (A) Immunofluorescence of the indicated proteins and detection of EGFP signal from the EGFP–MHF1 fusion protein transiently expressed in the cells for 48 h (no Triton pre-extraction). (B) Immunofluorescence of the indicated proteins 60 min after IR (4 Gy) and detection of EGFP signal from the EGFP–MHF1 fusion protein transiently expressed in the cells for 48 h (no Triton pre-extraction). (C) Immunofluorescence of the indicated proteins and detection of EGFP signal from the EGFP–MHF1 fusion protein transiently expressed in the cells for 48 h (with Triton pre-extraction). (D) Immunofluorescence of the indicated proteins 60 min after IR (4 Gy) and detection of EGFP signal from the EGFP–MHF1 fusion protein transiently expressed in the cells for 48 h (with Triton pre-extraction). (E–F) Immunofluorescence of the indicated proteins 60 min after IR (4 Gy) in the indicated siRNA-treated U2OS cells: (E) MHF1 and FANCI and (F) FANCI and γ-H2AX. (G) Immunofluorescence of CENPA, MHF1, and NBS1, as indicated, 60 min after mock treatment or IR (4 Gy). Note that anti-MHF1 does not detect kinetochore staining in Triton-X100 extracted cells. (H) FACS analysis of U2OS cells treated with the indicated siRNAs and incubated with nocodazole (100 ng/ml) or mock treated with DMSO. After 12 h cells were washed with 1× PBS and released in normal media for an additional 12 h. Cells positive for H3-pS10 mitotic marker were quantified at the indicated time points.
(TIF)

Table S1 List of Atm putative interactors identified by SILAC analyses from *Gallus gallus* total cell extracts. Protein IDs are from the IPI database and refer to chicken entries. The protein description provides a full name of the gene, and since the chicken database is not yet fully characterized and annotated, we provide in red a personal annotation resulting from manually inserting uniprot ID, NCBI ID, or IPI ID into respective search engines. R10/K8 is the heavy media used to label Atm-tagged cells, whereas R0/K0 is the normal media used as a blank; the numbers provided in columns C and D therefore represent the relative enrichment in pull-downs from cells grown in heavy media compared to the blank. For each hit, the number of peptides identified, the mass in kDa, the number of amino residues in the full-length protein identified, the confidence of identification expressed as Posterior Error Probability (PEP), and the intensity of the peaks read by the machine for Heavy (H) and Light (L) media are listed.
(XLSX)

## Abbreviations

DDR: DNA damage response
DSB: DNA double strand break
dsDNA: double-stranded DNA
FA: Fanconi anaemia
HR: homologous recombination
ICL: interstrand cross-links
IR: ionizing radiation
IRIF: IR induced foci
NHEJ: nonhomologous end joining
ssDNA: single-stranded DNA
TLS: translesion synthesis
