# Correction: The RSF1 Histone-Remodelling Factor Facilitates DNA Double Strand Break Repair by Recruiting Centromeric and Fanconi Anaemia Proteins

**DOI:** 10.1371/journal.pbio.1002595

**Published:** 2017-02-01

**Authors:** Fabio Pessina, Noel F. Lowndes

Fig 2 has been replaced with replicate data given uncertainties concerning the data of one of the western blots presented. The figure legend remains the same.

**Fig 2:**
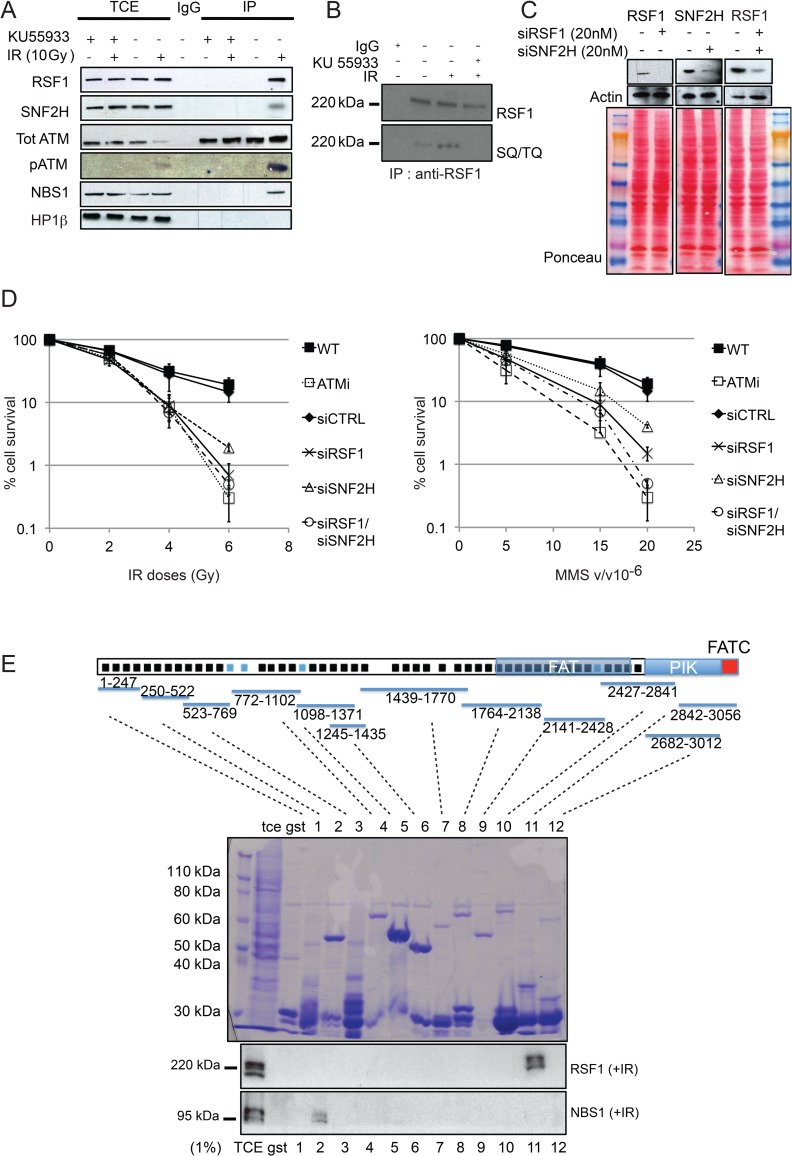
RSF1 is a novel ATM-interacting protein required for cell survival after DNA damage. (A) Co-immunoprecipitation (note that benzonase was included during cell lysis to solubilise chromatin-bound proteins; see Materials and Methods) of the indicated proteins with ATM. U2OS cells were either mock treated or treated with 10 Gy IR and harvested 1 h after irradiation. Where indicated the ATM inhibitor KU55933 was added directly to the media an hour before irradiation. (B) RSF1 immunoprecipitation. Cells were mock treated or treated with 5 Gy of IR and 5 Gy of IR plus ATM inhibitor. Elutions were blotted with RSF1 monoclonal antibody (Millipore) and general SQ/TQ antibody (Cell Signalling). (C) Typical efficiency of RSF1 and SNF2H depletion (siRNA) in U2OS cells. (D) Clonogenic survival after the indicated treatments of U2OS cells with IR or MMS. (E) Recombinant GST fusion proteins expressing fragments of ATM proteins were purified by *E*. *coli*, and approximately 5 mg of each GST fusion protein was used to incubate with cell lysates from HEK293 cells that had been previously irradiated with 5 Gy of IR and harvested 1 h post-IR. Elutions were blotted for RSF1 together with NBS1 as a control [37]. Schematic adapted from [43] shows heat repeats (black squares) conserved in the PIK family and heat repeats specific for ATM protein (blue squares); the domains are indicated, as are the regions where the GST fragments are mapping.

Fig 3 was incorrect as some images in panel B were mistakenly added from other blots. The authors have provided a corrected version here whereby all blots in panel B have been replaced. The figure legend remains the same.

**Fig 3:**
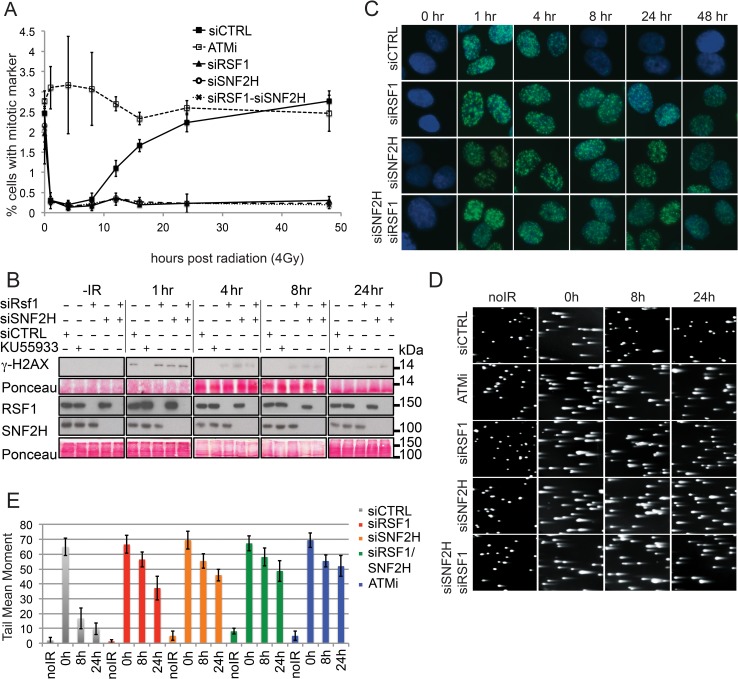
RSF1 promotes efficient DSB repair. (A) G2/M checkpoint analysis in U2OS cells. Inhibition of ATM or specific siRNA depletions are as indicated. Cells positive for the mitotic marker were followed for 48 h after irradiation. (B) Western blot analysis of c-H2AX phosphorylation after IR (4 Gy) over 24 h in U2OS cells treated as indicated. The extent of knockdown is indicated in the RSF1 and SNF2H blots. (C) Immunofluorescence showing c-H2AX IRIF formation and persistence in U2OS cells over 48 h postradiation (4 Gy) and treated with the indicated siRNAs. (D) Neutral comet assay showing the repair of fragmented DNA induced by 2 Gy of IR in U2OS cells treated either with an ATM inhibitor (KU55933) or with the indicated siRNAs. (E) Quantification of tail moments represented in the neutral comet assay shown in Fig 3D. Error bars indicate standard error of the mean (SEM) from three independent experiments.

Fig 6 has been replaced due to irregularities in the consistency of background signal for some images in panels A–F. The authors have provided a corrected version. The figure legend remains the same.

**Fig 6:**
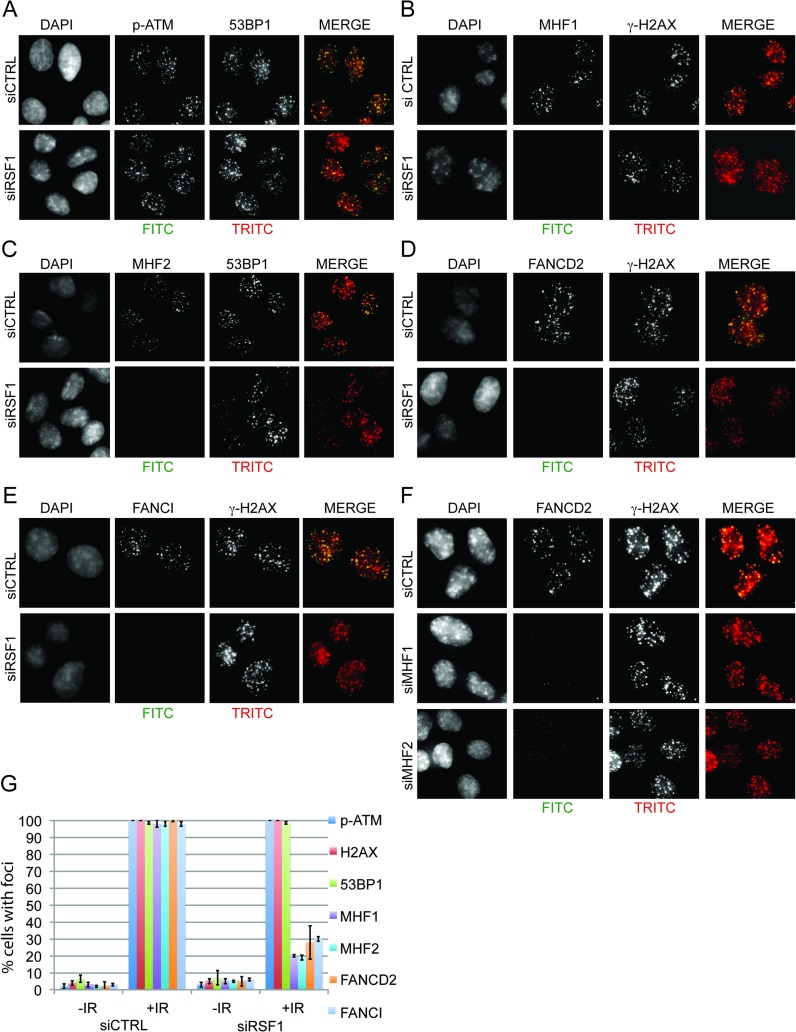
RSF1 is required for the recruitment of CENPS/MHF1, CENPX/MHF2, FANCD2, and FANCI to IRIF. (A–F) Immunofluorescence of the indicated proteins 60 min after IR (4 Gy) in the indicated siRNA-treated U2OS cells: (A) p-ATM and 53BP1, (B) MHF1 and c-H2AX, (C) MHF2 and 53BP1, (D) FANCD2 and c-H2AX, (E) FANCI and c-H2AX, and (F) FANCD2 and c-H2AX. (C) Quantification of pATM, c-H2AX, 53BP1, MHF1, MHF2, FANCI, and FANCD2 IRIF (when cells were depleted of RSF1). At least 100 cells were counted for each set of data; cells with more than 10 foci were considered positive. Error indicates SEM.
