# Expression of Concern: The RSF1 Histone-Remodelling Factor Facilitates DNA Double-Strand Break Repair by Recruiting Centromeric and Fanconi Anaemia Proteins

**DOI:** 10.1371/journal.pbio.3002635

**Published:** 2024-05-07

**Authors:** The PLOS Biology Editors

Following the publication and subsequent Correction of this article [1, 2], concerns were raised regarding results presented in Fig 6. Specifically, the Fig 6D siRSF1 panels appear to partially overlap with the Fig 6E siRSF1 panels.

The corresponding author stated that the FANCD2 + γH2AX files were inadvertently used to prepare the figure panels reporting both the FANCD2 and FANCI experiments, but indicated that the original data underlying this study are no longer available. In the absence of the underlying data, the Fig 6 concerns cannot be fully resolved.

In light of the concerns raised, the *PLOS Biology* Editors issue this Expression of Concern to notify readers of the above concerns and to inform readers that the Fig 6 results should be interpreted with caution.

The *PLOS Biology* Editors, as well as the authors, regret that the issue was not detected at the time Fig 6 was corrected in [2].

## References

[1] Pessina F, Lowndes NF (2014) The RSF1 Histone-Remodelling Factor Facilitates DNA Double-Strand Break Repair by Recruiting Centromeric and Fanconi Anaemia Proteins. PLoS Biol 12(5): e1001856. 10.1371/journal.pbio.1001856 24800743 PMC4011676

[2] Pessina F, Lowndes NF (2017) Correction: The RSF1 Histone-Remodelling Factor Facilitates DNA Double Strand Break Repair by Recruiting Centromeric and Fanconi Anaemia Proteins. PLoS Biol 15(2): e1002595. 10.1371/journal.pbio.1002595 28146553 PMC5287444

