# Supplementary figures and images for: The RSF1 Histone-Remodelling Factor Facilitates DNA Double-Strand Break Repair by Recruiting Centromeric and Fanconi Anaemia Proteins
Source: PLoS Biol. 2014 May 6;12(5):e1001856. doi: 10.1371/journal.pbio.1001856 (PMC4011676; doi:10.1371/journal.pbio.1001856)

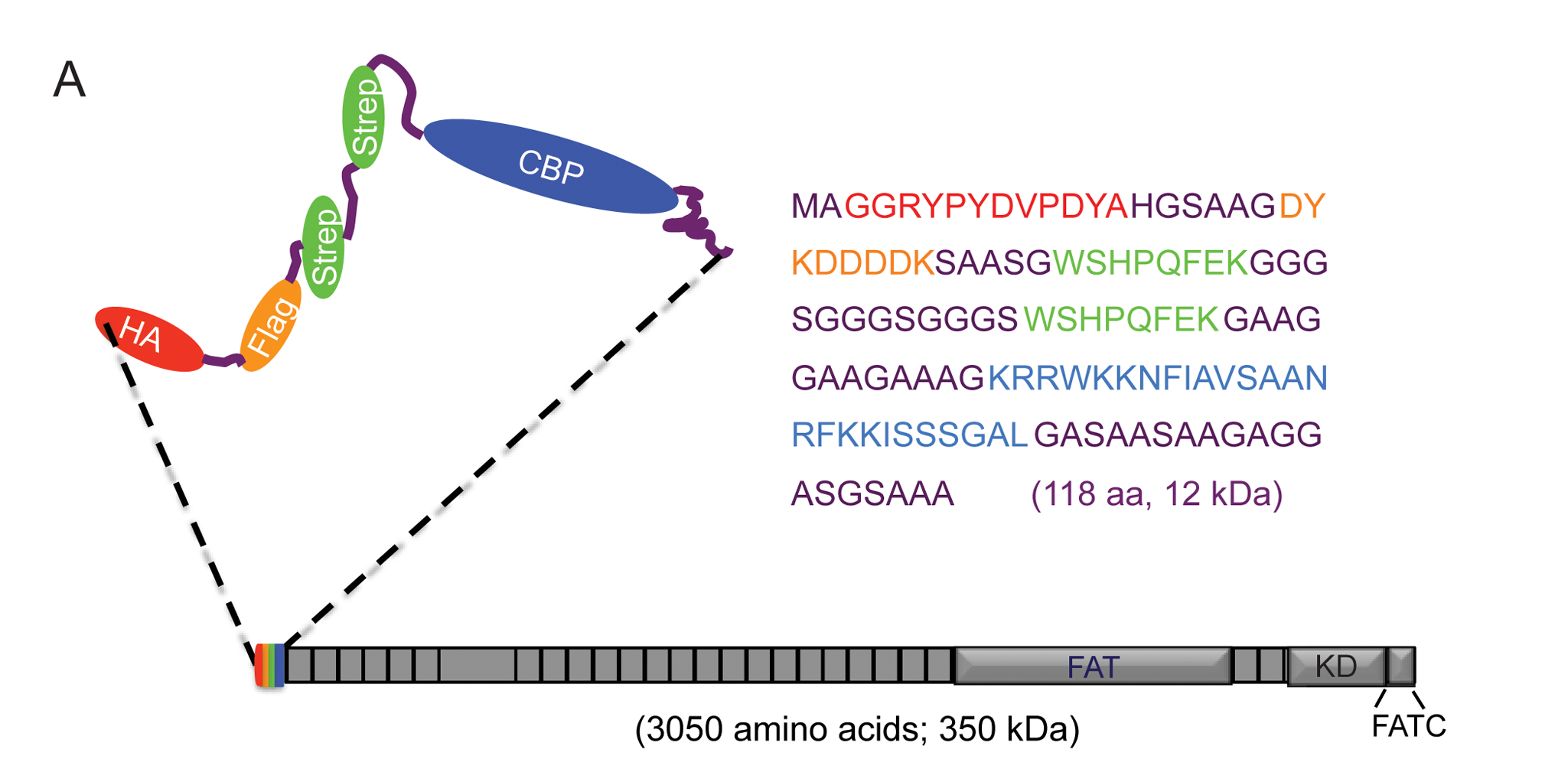

Supplement: Figure S1 — The HFSC-tag. (A) Schematic of HFSC–Atm and amino acid sequence of the HFSC-tag that encodes four tandem affinity purification epitopes (HA, Flag, Strep-tag II, and calmodulin binding protein) and a 19 amino acid linker to insulate the tag from the N-terminus of the tagged protein. (TIF) [file pbio.1001856.s001.tif]

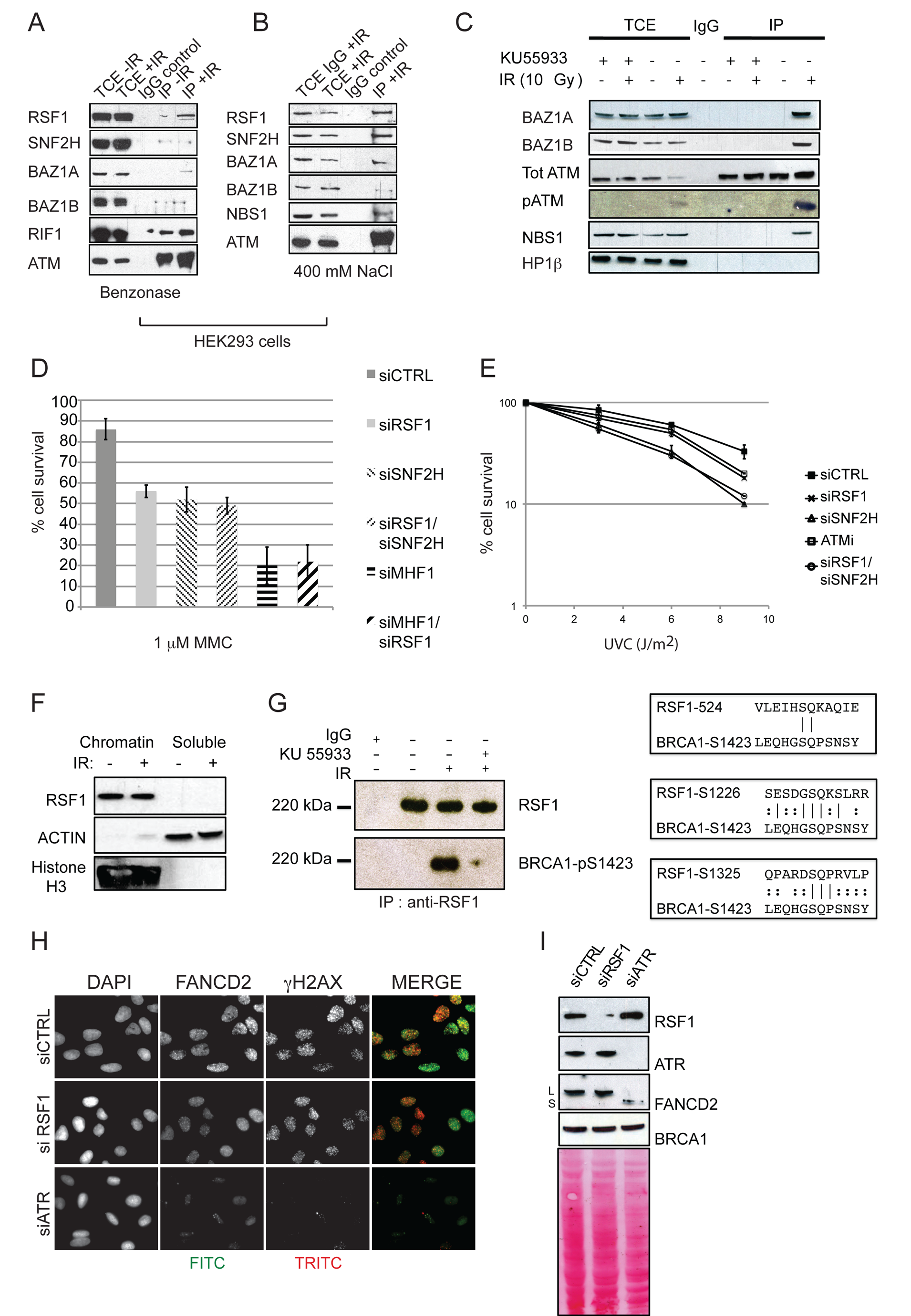

Supplement: Figure S2 — Analyses of ATM-interacting proteins using HEK293 cells and cell survival after MMC and UVC treatments. (A and B) Co-immunoprecipitation, using extracts prepared from HEK293 cells, of the indicated proteins with ATM. Chromatin bound proteins were solubilised by either benzonase (A) or, alternatively, 450 mM NaCl (B) treatments (see Materials and Methods) during cell lysis. HEK293 cells were either mock treated or treated with 10 Gy IR and harvested 1 h after irradiation. Where indicated the ATM inhibitor KU55933 was added directly to the media an hour before irradiation. (C) Co-immunoprecipitation from Figure 2A confirms, additionally, interaction of BAZ1A and BAZ1B proteins with ATM, using extracts prepared from U2OS cells. Blots of total ATM, pATM, NBS1, and HP1β are the same as in Figure 2A. Chromatin bound proteins were solubilised by benzonase treatment during cell lysis (see Materials and Methods). U2OS cells were either mock treated or treated with 10 Gy IR and harvested 1 h after irradiation. Where indicated the ATM inhibitor KU55933 was added directly to the media an hour before irradiation. (D and E) Survival of U2OS cells after treatment with MMC (D) or UVC (E) at the indicated doses and siRNA or ATMi (KU55933) treatments. Error bars indicate standard error of the mean (SEM) from three independent experiments. (F) Western blot analysis of the indicated proteins after chromatin fractionation of cell extracts prepared from U2OS cells. Note that in this assay, benzonase was added to the material pelleted after cell lysis (see Materials and Methods). (G) RSF1 immunoprecipitation. Cells were mock treated, treated with 5 Gy of IR and 5 Gy of IR plus ATM inhibitor, and harvested 1 h post-IR. Elutions were blotted with the RSF1 monoclonal antibody (Millipore) and anti–BRCA1-pS1423. The schematic to the right shows an alignment of BRCA1-S1423 with the three consensus PIK kinase sites of RSF1. (H) Immunofluorescence of the FANCD2 and γ-H2AX proteins after 24 h incu [file pbio.1001856.s002.tif]

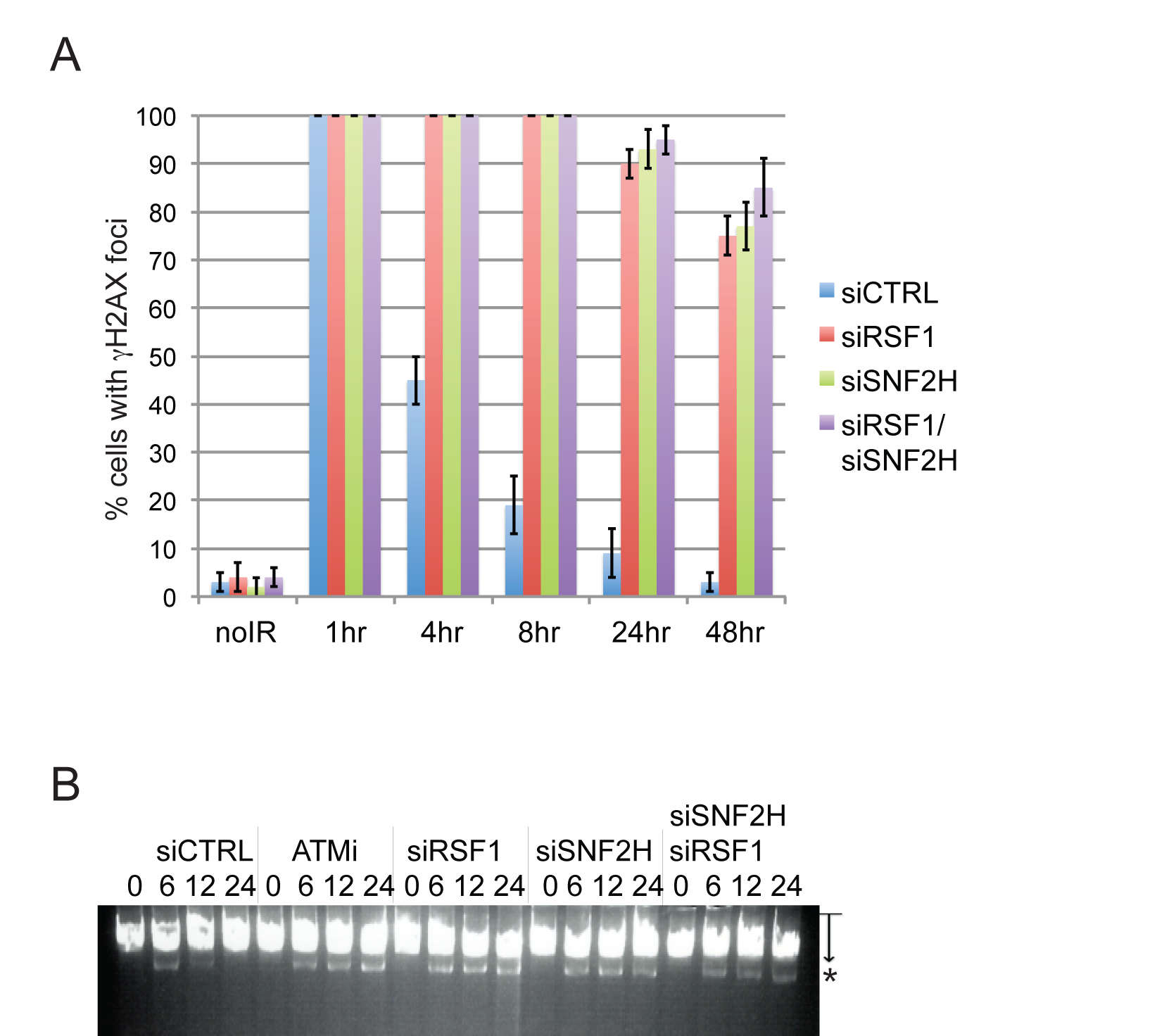

Supplement: Figure S3 — Quantification of γH2AX foci and pulse-field gel analysis of DSB repair. (A) Quantification of γH2AX IRIF cells represented in Figure 2C. Cells with greater than 10 γH2AX IRIF were scored as positive. Error bars indicate standard error of the mean (SEM) from three independent experiments. (B) Analysis of fragmented DNA after IR (10 Gy) by pulse-field gel electrophoresis. Time postirradiation is indicated in hours. Also indicated are the respective siRNA or ATMi (KU55933) treatments. The asterisk indicates the fragmented DNA detected under the electrophoretic conditions used. (TIF) [file pbio.1001856.s003.tif]

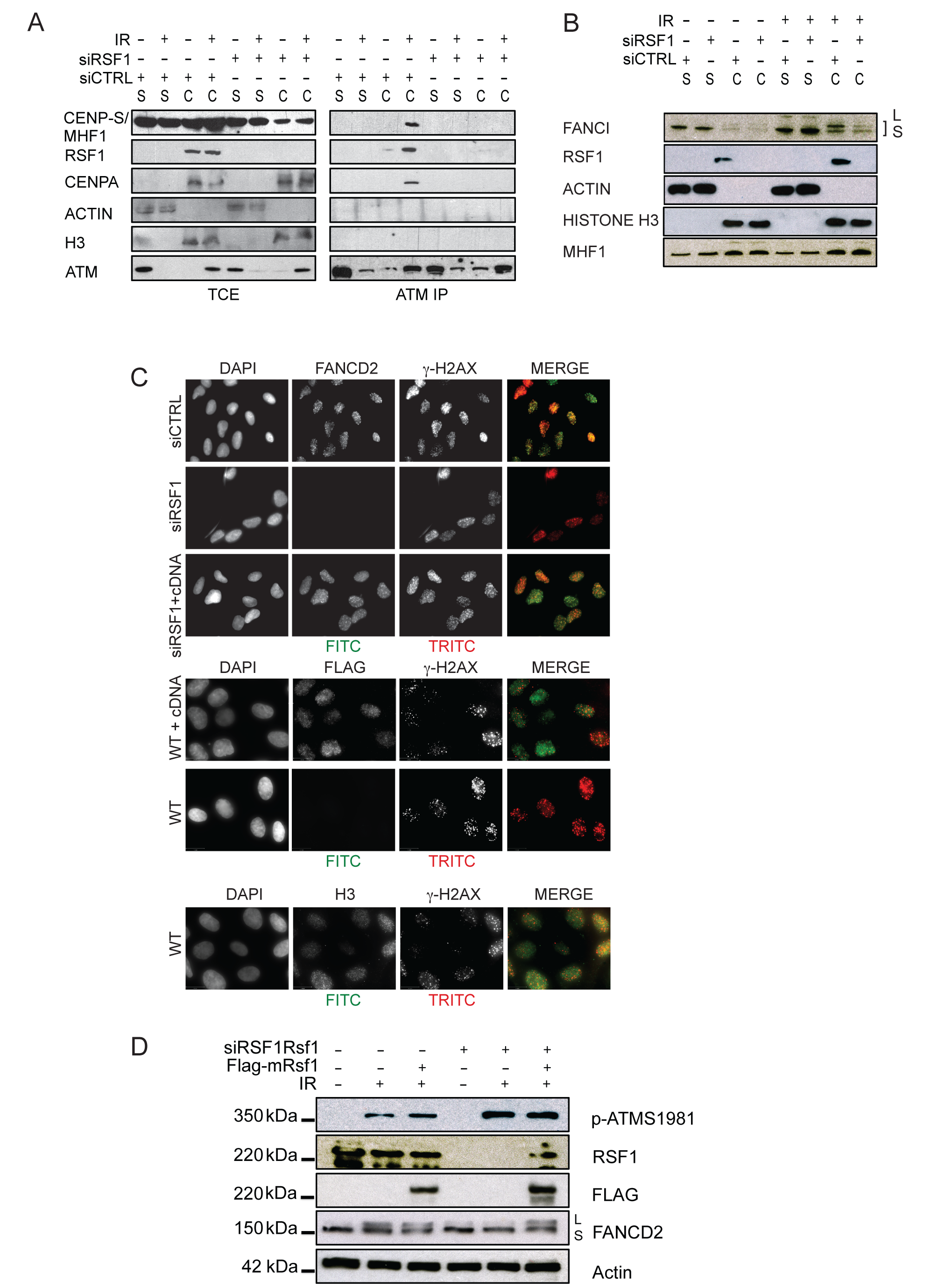

Supplement: Figure S4 — The RSF complex promotes DSB repair and interacts with centromeric proteins. (A) Co-immunoprecipitation of the indicated proteins from U2OS cells with ATM from the soluble and chromatin fraction. Note that the chromatin was solubilized by benzonase treatment. The cells were cross-linked (1% PFA treatment for 10 min at room temperature) prior to harvesting and were either mock treated or irradiated (10 Gy). (B) Western blot analysis of the indicated proteins after chromatin fractionation of cell extracts prepared from U2OS cells after the indicated treatments (IR was 4 Gy and siRNAs were as indicated). S and C refer to soluble and chromatin fraction, respectively. (C) Immunofluorescence of FANCD2 and γ-H2AX 1 h after IR (4 Gy) in the indicated siRNA-treated U2OS cells. Formation of FANCD2 IRIF is rescued by expression of Flag-tagged mouse Rsf1 in cells in which endogenous human RSF1 has been depleted. (D) Western blotting showing successful expression of Flag-tagged mouse Rsf1 in U2OS cells. Cells depleted of endogenous human RSF1 expressing Flag-mRsf1 display normal levels of mono-ubiquitination of FANCD2. (TIF) [file pbio.1001856.s004.tif]

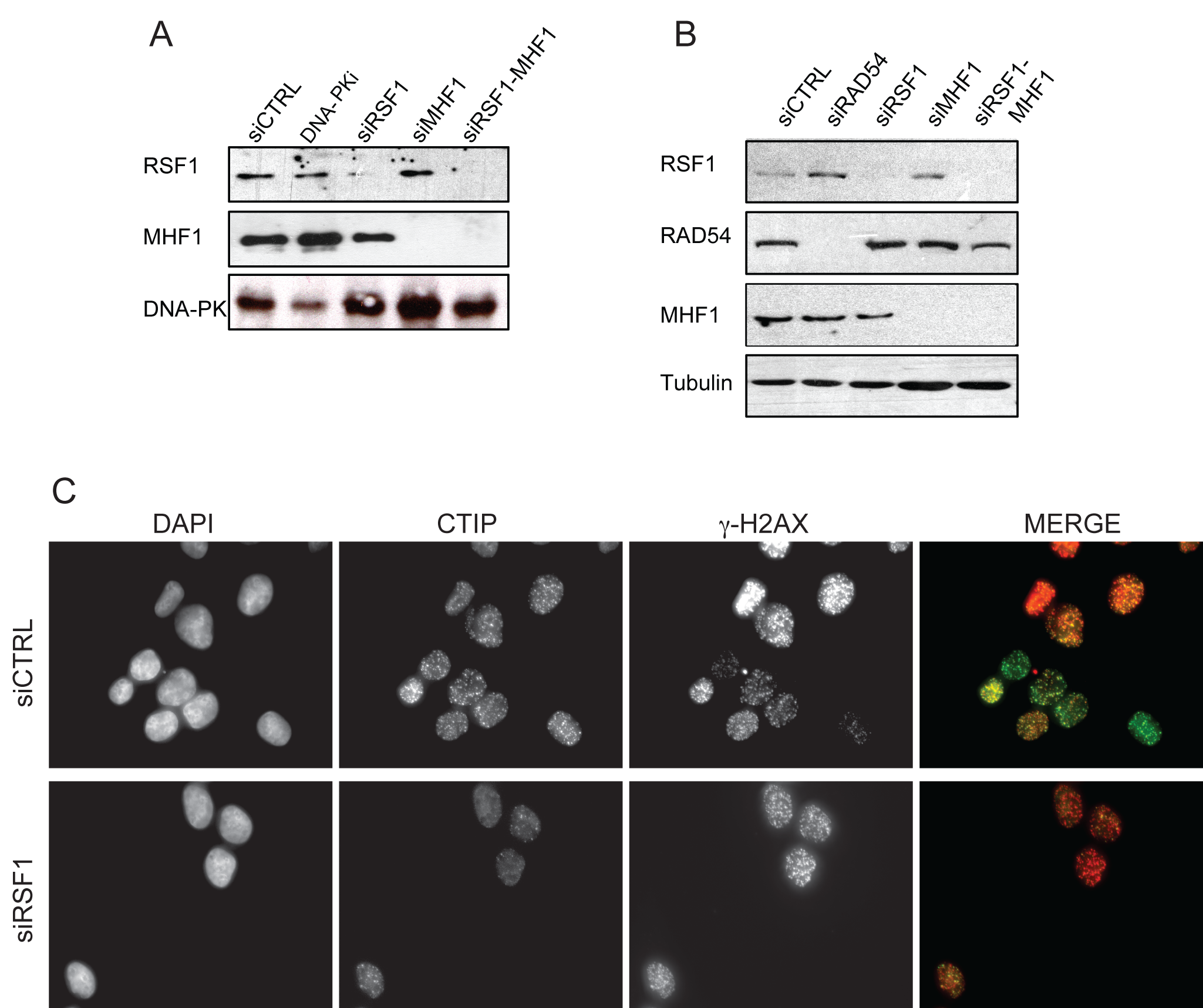

Supplement: Figure S5 — Efficiency of RSF1, CENPS/MHF1, and RAD54 depletion. (A and B) Typical knockdown efficiency of siRNA used for NHEJ (A) and HR (B) assays. (C) Immunofluorescence of CtIP and γ-H2AX 3 h after IR (4 Gy) in the indicated siRNA-treated U2OS cells. (TIF) [file pbio.1001856.s005.tif]

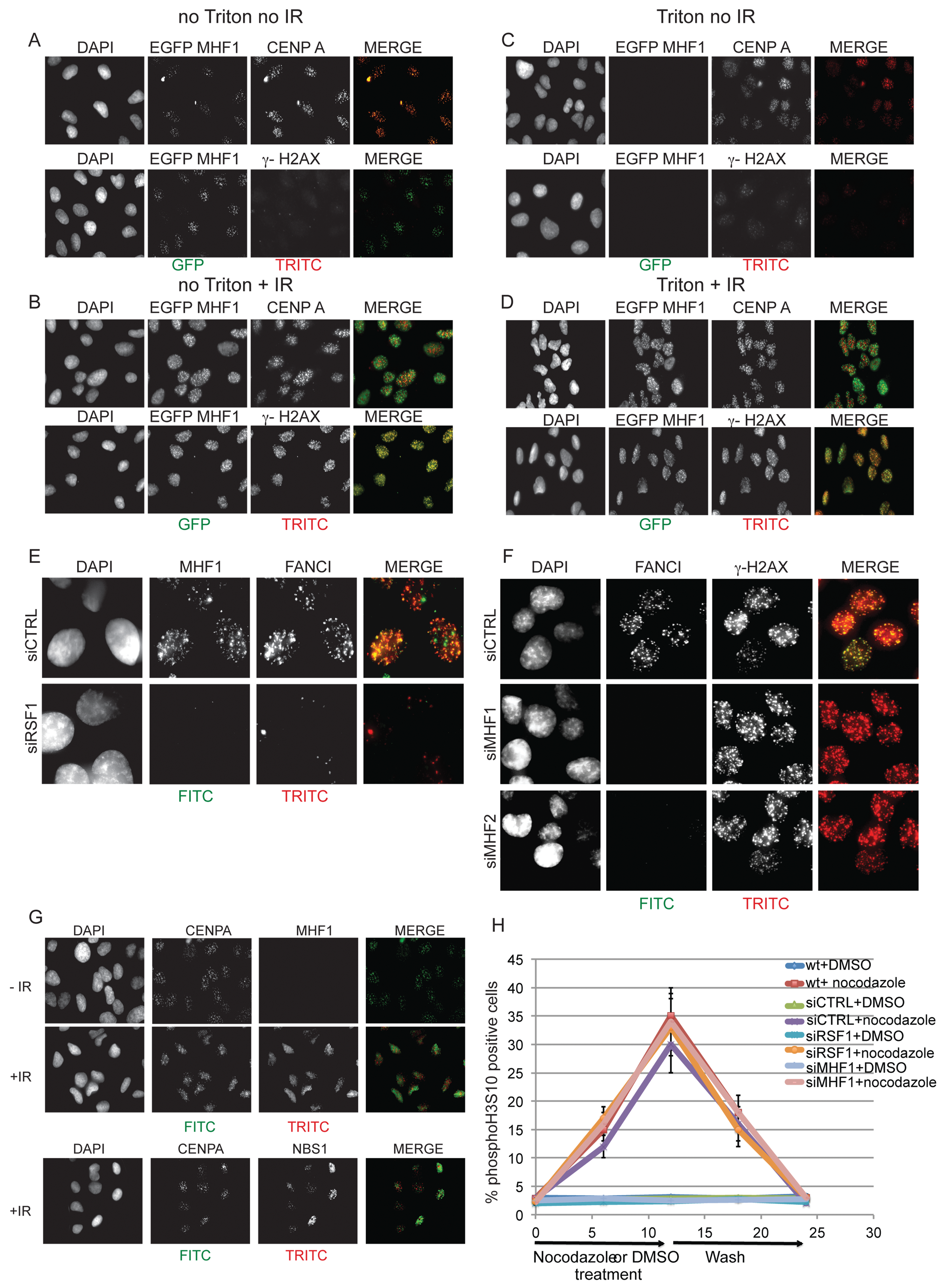

Supplement: Figure S6 — RSF1 regulates FANCI through the centromeric protein MHF1. (A) Immunofluorescence of the indicated proteins and detection of EGFP signal from the EGFP–MHF1 fusion protein transiently expressed in the cells for 48 h (no Triton pre-extraction). (B) Immunofluorescence of the indicated proteins 60 min after IR (4 Gy) and detection of EGFP signal from the EGFP–MHF1 fusion protein transiently expressed in the cells for 48 h (no Triton pre-extraction). (C) Immunofluorescence of the indicated proteins and detection of EGFP signal from the EGFP–MHF1 fusion protein transiently expressed in the cells for 48 h (with Triton pre-extraction). (D) Immunofluorescence of the indicated proteins 60 min after IR (4 Gy) and detection of EGFP signal from the EGFP–MHF1 fusion protein transiently expressed in the cells for 48 h (with Triton pre-extraction). (E–F) Immunofluorescence of the indicated proteins 60 min after IR (4 Gy) in the indicated siRNA-treated U2OS cells: (E) MHF1 and FANCI and (F) FANCI and γ-H2AX. (G) Immunofluorescence of CENPA, MHF1, and NBS1, as indicated, 60 min after mock treatment or IR (4 Gy). Note that anti-MHF1 does not detect kinetochore staining in Triton-X100 extracted cells. (H) FACS analysis of U2OS cells treated with the indicated siRNAs and incubated with nocodazole (100 ng/ml) or mock treated with DMSO. After 12 h cells were washed with 1× PBS and released in normal media for an additional 12 h. Cells positive for H3-pS10 mitotic marker were quantified at the indicated time points. (TIF) [file pbio.1001856.s006.tif]
